# Cell Wall Matrix Polysaccharides Contribute to Salt–Alkali Tolerance in Rice

**DOI:** 10.3390/ijms232315019

**Published:** 2022-11-30

**Authors:** Zhijian Liu, Yongzhi Hu, Anping Du, Lan Yu, Xingyue Fu, Cuili Wu, Longxiang Lu, Yangxuan Liu, Songhu Wang, Weizao Huang, Shengbin Tu, Xinrong Ma, Hui Li

**Affiliations:** 1 Chengdu Institute of Biology, Chinese Academy of Sciences, Chengdu 610041, China; 2 University of Chinese Academy of Sciences, Beijing 100049, China; 3 College of Ecology and Environment, Chengdu University of Technology, Chengdu 610059, China

**Keywords:** salt–alkali stress, cell wall matrix polysaccharides, hemicellulose, *OsCSLD4*, pectin, grain yield, rice

## Abstract

Salt–alkali stress threatens the resilience to variable environments and thus the grain yield of rice. However, how rice responds to salt–alkali stress at the molecular level is poorly understood. Here, we report isolation of a novel salt–alkali-tolerant rice (SATR) by screening more than 700 germplasm accessions. Using 93-11, a widely grown cultivar, as a control, we characterized SATR in response to strong salt–alkali stress (SSAS). SATR exhibited SSAS tolerance higher than 93-11, as indicated by a higher survival rate, associated with higher peroxidase activity and total soluble sugar content but lower malonaldehyde accumulation. A transcriptome study showed that cell wall biogenesis-related pathways were most significantly enriched in SATR relative to 93-11 upon SSAS. Furthermore, higher induction of gene expression in the cell wall matrix polysaccharide biosynthesis pathway, coupled with higher accumulations of hemicellulose and pectin as well as measurable physio-biochemical adaptive responses, may explain the strong SSAS tolerance in SATR. We mapped SSAS tolerance to five genomic regions in which 35 genes were candidates potentially governing SSAS tolerance. The 1,4-β-D-xylan synthase gene *OsCSLD4* in hemicellulose biosynthesis pathway was investigated in details. The *OsCSLD4* function-disrupted mutant displayed reduced SSAS tolerance, biomass and grain yield, whereas the *OsCSLD4* overexpression lines exhibited increased SSAS tolerance. Collectively, this study not only reveals the potential role of cell wall matrix polysaccharides in mediating SSAS tolerance, but also highlights applicable value of *OsCSLD4* and the large-scale screening system in developing SSAS-tolerant rice.

## 1. Introduction

Saline–alkali soils account for about one-fifth of cultivated land in the world (about 1128 Mha), and the area is increasing by about 10% per year. Half of the arable land will be affected by soil salinization by 2050 [1,2,3]. Saline–alkali soils are classified into three groups including saline soils, sodic soils and saline-sodic soils. The calcium, magnesium and sodium are the primary cations in saline soils (pH < 8.5), Na_2_CO_3_ and NaHCO_3_ are the main salts in sodic soils (pH = 8.5–10.0), and saline-sodic soils (pH > 8.5) are the transitional group between saline soils and sodic soils [2]. Saline–alkali stress affects the growth, development, survival and yield of plants worldwide, including rice. Therefore, studying the regulatory mechanisms of rice plants adapting to saline–alkali stress and developing saline–alkali-tolerant rice cultivars are helpful for increasing the rice yield.

Salt stress is usually combined with alkali stress together in nature, forming combined salt–alkali stress (CSAS). Hence, CSAS contains multiple stresses, which mainly consists of ionic stress, high pH stress, osmotic stress and oxidative stress. In fact, most reported CSAS tolerance mechanisms in plants are similar to salt stress or alkali stress tolerance mechanisms, such as accumulating organic acids to bring down the pH around the roots and inhibit the precipitation of metal ions and phosphate [4,5], accumulating more osmolytes (proline, soluble sugars, betaine and soluble proteins) to adjust osmosis balance [6], increasing the activity of antioxidant enzymes (catalase, superoxide dismutase, peroxidase and ascorbate peroxidase) to detoxify reactive oxygen species (ROS) [6,7,8], and regulating stomatal closure through ABA-induced pathway [9,10]. Many related studies for saline–alkali stress are performed using NaHCO_3_ stress on the species including *Salix linearistipularis*, *Chlorella vulgaris*, *Arabidopsis thaliana*, *Glycine soja* and *Medicago sativa* [11,12,13,14,15,16,17,18]. However, quite a few studies were focused on CSAS in monocots such as *Puccinellia tenuiflora*, sorghum and rice [8]. *Puccinellia tenuiflora* is a gramineous monocot with strong CSAS tolerance. Compared with rice, *Puccinellia tenuiflora* can absorb more nutrients, maintain higher restriction of Na^+^ and H^+^ flux under CSAS [19]. The ABA-induced pathway is also involved in the CSAS response in sorghum [20]. In addition, overexpression of the *OsCu/Zn-SOD* gene in rice can enhance CSAS resistance through promoting ROS scavenging [21].

On the other hand, cell wall and other extracellular matrix including cutin and cuticular waxes are also critical for plants to respond to salt, alkali and CSAS. Cellulose, hemicellulose, pectin as well as lignin and suberin in some cases are essential components of plant cell wall, which are combined organically, forming the most vital apoplast barrier to protect root cells from abiotic stresses [22]. Thereinto, hemicellulose and pectin are often linked by covalent bonds, forming cell wall matrix polysaccharides and conferring the elasticity and plasticity to the cell wall [23,24,25]. Cellulose, lignin and suberin are embedded in cell wall, strengthening its mechanical properties [26,27,28]. Seed hemicelluloses can tailor mucilage properties and modulate salt tolerance in *Arabidopsis* [29]. Deposition of lignin and suberin in root cell wall can positively adjust salt tolerance against salt stress through limiting intakes of toxic ions (e.g., Na^+^) and reducing water loss in plants [30]. Meanwhile, cutin and cuticular waxes, which form extracellular lipid structure deposited over the shoot surfaces of land plants, can also enhance the tolerance of plants to salt stress by reducing the non-stomatal water loss in leaves [31,32,33]. In addition, microtubule, a critical component of cytoskeleton system, is not only involved in cell wall biogenesis, but also positively modulates the tolerance of plants against salt stress [34,35].

Cutin, suberin and cuticular waxes are long chain fatty acids (VLCFs) or their derivatives. Their biosynthesis pathways are mainly composed of type II fatty acid biosynthesis (FAS) in endoplasmic reticulum (ER), fatty acid elongation (FAE) in mitochondria, FAE in ER and ‘cutin, suberin and wax biosynthesis’ [31,36]. On the other hand, the hemicellulose and pectin biosynthesis pathway mainly starts with UDP-Glc (UDP-glucose). The conversion of UDP-Glc into pectin requires three-step catalytic reactions catalyzed by three groups of enzymes [UDP-glucose dehydrogenase (UG6D), UDP-glucuronate 4-epimerase (UGUE) and α-1,4-galacturonosyltransferase (GAUT)] (https://www.kegg.jp/kegg-bin/show_pathway?dosa00520) (accessed on 20 October 2022). The conversion of UDP-Glc into 1,4-β-D-xylan requires three-step catalytic reactions catalyzed by four groups of enzymes [UG6D, UDP-apiose/xylose synthase (AXS), UDP-glucuronate decarboxylase (UXS) and 1,4-β-D-xylan synthase (OsCSLD4)]. In the hemicellulose biosynthesis pathway, the 1,4-β-D-xylan synthase (EC:2.4.2.24) is encoded only by *OsCSLD4* (*LOC_Os12g36890*) gene, which is responsible for catalyzing UDP-D-xylose into 1,4-β-D-xylan. The corresponding product, 1,4-β-D-xylan is a major hemicellulose in primary and secondary cell walls of gramineous plants [37]. *OsCSLD4* was previously reported to play important roles in cell wall biosynthesis, plant growth and stress response in rice. The *OsCSLD4* mutant *nd1* had reduced xylan and cellulose contents in rice culms and exhibited some growth defects including stunted growth, narrow and rolled leaves [38]. Recently, *OsCSLD4* was reported to play an important role in the response of rice to salt stress by mediating abscisic acid biosynthesis to regulate osmotic stress tolerance. The *nd1* mutant displayed decreased tolerance to salt and osmotic stress, but the *OsCSLD4* overexpression lines showed improved salt tolerance [39]. However, whether *OsCSLD4* and other cell wall matrix polysaccharide biosynthesis genes regulate the tolerance of rice plants against salt–alkali stress is still unknown.

Thus, it is necessary to screen rice germplasms tolerant to salt–alkali stress and further uncover the underlying regulatory mechanisms. In this study, we isolated and characterized a novel salt–alkali-tolerant rice germplasm, SATR, from more than 700 rice accessions by constructing and using a large-scale screening system. We preliminarily revealed the mechanism that induced accumulation of cell wall matrix polysaccharides controlled by the biosynthesis pathway genes including *OsCSLD4*, and adaptive physio-biochemical responses modulating salt–alkali tolerance in rice. Therefore, this study also highlighted the potentiality and value of *OsCSLD4* and other cell wall matrix polysaccharide biosynthesis genes in molecular breeding to develop high-yield and salt–alkali-tolerant rice.

## 2. Results

### 2.1. Salt–Alkali Tolerance of SATR Is Conferred by Adaptive Physio-Biochemical Responses

By constructing and using a large-scale screening system for obtaining salt–alkali-tolerant rice germplasms, we firstly screened 10 relatively strong tolerant rice germplasms from more than 700 accessions. A novel strongest combined salt–alkali-tolerant rice germplasm (SATR) was finally isolated from the 10 candidate accessions by repetitive evaluation and identification (Figures S1D and S2). We further identified the tolerance of SATR against single salt stress, single alkali stress and combined salt–alkali stress, by using the 93-11 cultivar as a control. Both SATR and 93-11 seedlings showed similar adaptive phenotypes under single salt or alkali stress with normal growth and green leaves, except for some yellow leaves or leaf tips (Figure S2A–C).

**Figure 1:**
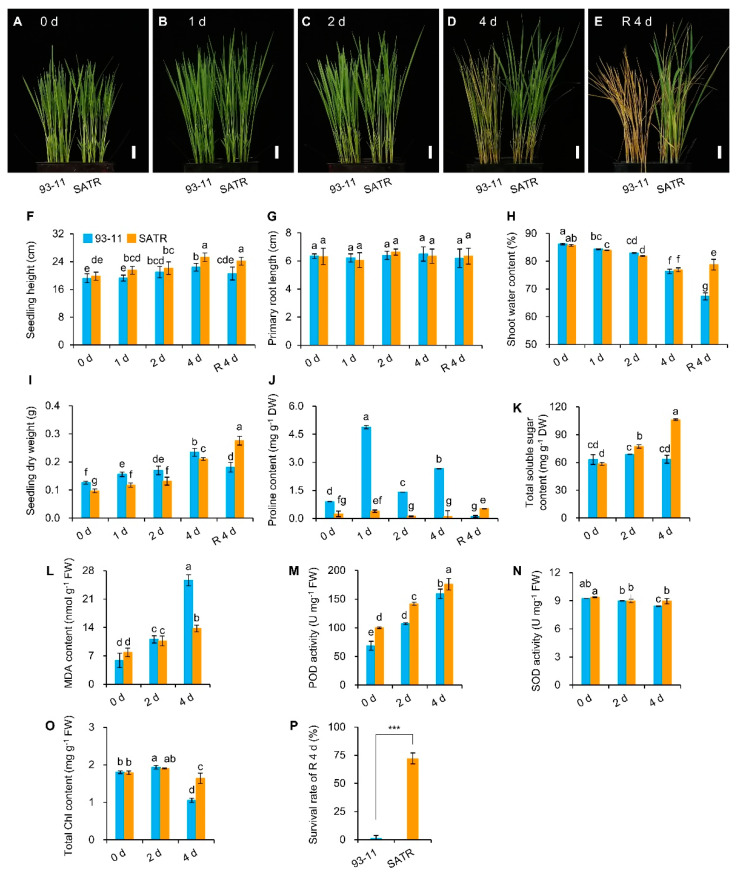
Phenotypic and physio-biochemical analysis in SATR and 93-11 seedlings under SSAS. (**A**–**E**) Seedlings of 93-11 and SATR before SSAS (**A**), on the 1st (**B**), 2nd (**C**) and 4th (**D**) days after SSAS treatment, and 4 days of recovery (**E**), respectively. (**F**–**P**) Comparisons of phenotypic and physio-biochemical indexes, including seedling height (**F**), primary root length (**G**), shoot water content (**H**), seedling dry weight (**I**), as well as proline content (**J**), total soluble sugar content (**K**), malonaldehyde (MDA) content (**L**), peroxidase (POD) activity (**M**), superoxide dismutase (SOD) activity (**N**) and total chlorophyll content (**O**) of shoots in SATR and 93-11 before SSAS, under SSAS and recovery after SSAS, respectively. (**P**) Comparison of the survival rate between SATR and 93-11 seedlings after 4 days of recovery. d, day or days; R 4 d, after 4 days of recovery. Bar = 2 cm (**A**–**E**). The blue and orange bars indicate 93-11 and SATR, respectively (**F**–**P**). Data is given as mean ± SD, which was obtained from three independent biological replicates (**F**–**P**), and each replicate contained 5 seedlings (**F**–**O**) or 24 seedlings (**P**). Chl, chlorophyll. The different letter on the bars in each bar chart indicates the significant difference at *p* < 0.05 level obtained by one-way analysis of variance (ANOVA) and Duncan’s new multiple range test (**F**–**O**). *** *p* < 0.001, Student’s *t*-test (**P**).

However, SATR and 93-11 seedlings exhibited obvious differences in tolerance against combined salt–alkali stress. Firstly, both 93-11 and SATR seedlings grew normally on the 0th, 1st and 2nd days after SSAS treatment. The 93-11 seedlings on the 3rd and 4th days after SSAS treatment showed susceptible phenotypes with severely wilted leaf blades and wizened leaf tips. However, SATR seedlings were less affected by SSAS treatment with relatively normal growth and slightly yellow or wizened leaf tips. Moreover, the difference in tolerance between SATR and 93-11 seedlings became greater and more obvious after 4 or 5 days of recovery. SATR displayed much more living and green seedlings than 93-11 indicating a much higher survival rate (72.22% and 1.39% for SATR and 93-11, respectively) (Figure S2D,E and Figure 1A–E). These results indicate that although both SATR and 93-11 seedlings are tolerant to single salt or alkali stress, SATR is a rice germplasm with strong tolerance against combined salt–alkali stress at seedling stage than the susceptible control, 93-11. 

To investigate the internal causes of SATR and 93-11 seedlings which resulted in different SSAS tolerance between them, some representative physio-biochemical parameters were determined in their seedlings under SSAS. Both of their seedlings had decreased total chlorophyll contents on the 4th day after SSAS treatment compared with the 0th and 2nd days. However, the total chlorophyll contents were significantly higher in SATR shoots than that of 93-11 on the 4th day after SSAS treatment (Figure 1O). It is consistent with the greener leaves in SATR than 93-11 under SSAS.

Both SATR and 93-11 seedlings showed fluctuant and insignificantly different length of primary root after SSAS (Figure 1G). SATR had higher seedling height than 93-11 on the 1st and 4th days after SSAS treatment as well as 4 days of recovery (Figure 1F). Moreover, both of them showed a continuous decline in shoot water content from the 0th to 4th days after SSAS treatment. However, the SATR seedlings maintained significantly higher water content after 4 days of recovery after SSAS (Figure 1H). It is consistent with the result that SATR seedlings displayed less and slighter wilted leaves than 93-11 under SSAS and after recovery (Figure 1B–E and Figure S2D,E). On the other hand, it showed consistent changing curves in the biomass related parameters including shoot diameter, shoot fresh weight, shoot dry weight, root dry weight and seedling dry weight between SATR and 93-11 seedlings after SSAS and recovery. As a whole, compared with the slow increase in these biomass-related parameters in 93-11 seedlings from the 0th to 4th day after SSAS treatment, versus a subsequent sharp decrease to 4 days of recovery, the SATR seedlings exhibited a continual increase in these biomass-related parameters, which are significantly higher in SATR than 93-11 seedlings after 4 days of recovery. Thereinto, SATR had a 35.77%, 126.66%, 47.45%, 87.15% and 52.16% higher shoot diameter, shoot fresh weight, shoot dry weight, root dry weight and seedling dry weight, respectively, than that of 93-11 after 4 days of recovery after SSAS. It further reveals that SATR maintained better growth than 93-11 under SSAS, indicated by a continually increasing biomass after SSAS and higher biomass of SATR seedlings after 4 days of recovery (Figure S3A–D and Figure 1I).

Both SATR and 93-11 accumulated a more than 60-fold higher total soluble sugar content compared to proline content from 0 day to 4 days of recovery, on average. Furthermore, SATR accumulated higher total soluble sugar contents, with a 12.44% and 67.02% increase on the 2nd and 4th day after SSAS treatment, respectively, as compared to 93-11 (Figure 1J,K). Additionally, SATR seedlings maintained remarkably lower malondialdehyde content on the 4th day after SSAS treatment than 93-11 (Figure 1L), which suggests that SATR seedlings were subjected to less oxidative damage than 93-11 under SSAS. Correspondingly, significantly stronger POD activity on the 0th, 2nd and 4th day after SSAS treatment, as well as significantly stronger SOD activity on the 4th day after SSAS treatment, were detected in SATR seedlings as compared to 93-11 (Figure 1M,N).

Taken together, the combined salt–alkali stress tolerance of SATR is attributed to adaptive physio-biochemical regulation processes including detoxification of osmotic and oxidative stresses. Thus, SATR is an ideal germplasm for studying the molecular mechanisms of combined salt–alkali stress tolerance in rice.

### 2.2. Enrichment of the Highlighted Cell Wall Related GO Terms and KEGG Pathways between SATR and 93-11 under SSAS

To investigate the gene expressions and regulatory networks which modulate salt–alkali tolerance of SATR, we performed a comparative transcriptome study including Gene Ontology (GO) and KEGG enrichment analysis between SATR and 93-11 seedlings under SSAS for 0 days, 2 days and 4 days. The RNA-seq data had sufficient and high-quality reads, high mapping percentages of clean reads to the reference genome, and good repeatability within independent biological replicates as a whole (Tables S1 and S2; Figure S4A,B), which can be used for the subsequent differentially expressed genes (DEGs) analysis. The total DEGs slightly increased from 4093 on the 0th day to 4598 on the 2nd day, but sharply increased to 8671 on the 4th day (Figure S5A–C; Dataset S1). It coincides with the result that SATR and 93-11 showed much more obvious differences in SSAS tolerance and correspondingly physio-biochemical parameters on the 2nd and 4th days than that on the 0th day. 

By GO enrichment analysis, GO terms of oxidation–reduction activities and some abiotic stress-related processes were significantly enriched from downregulated DEGs between SATR and 93-11 on the 2nd and 4th day after SSAS treatment. In addition, cell wall and cytoskeleton system related GO terms were significantly enriched from the upregulated DEGs between SATR and 93-11 on the 4th day after SSAS treatment (Figures S6 and S7). By further KEGG enrichment analysis, the DNA repair related pathways were significantly enriched from the upregulated DEGs on the 2nd day after SSAS treatment. More importantly, the cell wall matrix polysaccharide biosynthesis-related pathways including cutin, suberin and wax biosynthesis were the significantly enriched KEGG pathways from the upregulated DEGs on the 4th day after SSAS treatment (Figure S8).

### 2.3. SATR Had Higher Expression Levels of Hemicellulose and Pectin Biosynthesis Pathway and Tubulin Genes under SSAS

**Figure 2:**
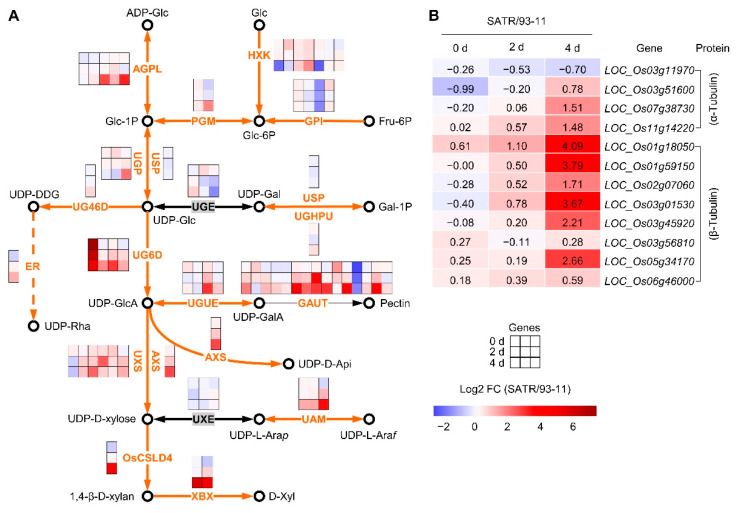
Expression analysis of hemicellulose and pectin biosynthesis pathway genes (**A**) and tubulin genes (**B**) between SATR and 93-11 under SSAS. The solid and dotted arrows indicate the direction of directly and indirectly enzyme-catalyzed reaction, respectively. The single-headed and double-headed arrows indicate irreversible and reversible reactions, respectively. The open circle indicates metabolite (substrate or product of enzyme reaction). The black symbol beside the metabolite indicates name of the corresponding metabolite. The orange name between two metabolites represents a group of enzymes involved in this reaction. The names of enzymes or metabolites were indicated with acronym. The color of small inner cell in the first, second and third row of the big composite box beside the enzyme corresponds to the value of log2 fold change (SATR/93-11) of the corresponding encoding gene between SATR and 93-11 seedling samples under SSAS for 0 days, 2 days and 4 days, respectively. The orange arrows indicate the overall increased gene expressions of the encoding enzymes which catalyze the corresponding step of enzyme-catalyzed reaction in SATR compared with 93-11 (SATR/93-11) under SSAS. The detailed information of these enzyme-encoding genes and full names of the metabolites were summarized in Table S3, respectively. The thresholds for DEGs are |log2 [fold change (SATR/93-11)]| > 0.5850 (1.5-fold change value of gene expressions in SATR compared with 93-11) and *p*-value < 0.05.

To explore the roles of hemicellulose and pectin biosynthesis pathway to the SSAS tolerance of SATR seedlings, we further analyzed the gene expression levels between SATR and 93-11 in this pathway. A total of 67 enzyme-encoding genes, which encode the corresponding 21 groups of enzymes, are involved in 19 steps of catalyzed reactions in this pathway (Figure 2A). With the cutoff of |log2 [fold change (SATR/93-11)]| > 0.5850 (1.5-fold change value of gene expressions in SATR compared with 93-11) and *p*-value < 0.05, 7.46%, 11.94% and 80.60% genes showed significantly higher, significantly lower and insignificant change in expression levels in SATR, respectively, compared with 93-11 before SSAS (under SSAS for 0 day). Nevertheless, with the increase in SSAS time, the percentages of significantly higher expression genes continually increased from 7.46% to 17.91% and 49.25% on the 0th, 2nd and 4th day after SSAS treatment, respectively. Thereinto, excepting for the encoding genes of USP, UGE and UXE enzymes, expression levels of most hemicellulose and pectin biosynthetic genes (71.74% genes) were markedly higher in SATR seedlings on the 4th day after SSAS treatment, including the encoding genes of AGPL, PGM, UG46D, UGHPU, ER, UG6D, UGUE, GAUT, UXS, AXS, UAM, OsCSLD4 and XBX enzymes. 

These results indicate that SATR seedlings had higher gene expression levels in hemicellulose and pectin biosynthesis pathway induced by SSAS.

On the other hand, the ‘phagosome’ that includes tubulin proteins was the third most significantly enriched KEGG pathway from the upregulated DEGs between SATR and 93-11 on the 4th day after SSAS treatment (Figure S8A). Previous studies reported that microtubule is involved in cell wall organization and formation including matrix polysaccharides, as well as salt tolerance [34,35]. Thus, we further analyzed the expression levels of the four α-tubulin and the other eight β-tubulin encoding genes between SATR and 93-11 seedlings under SSAS (Figure 2B). Overall, compared with the 0th day, the α-tubulin and β-tubulin genes showed continually and increasingly higher expression levels in SATR than 93-11 on the 2nd and 4th day after SSAS treatment. A total of 75.00% α-tubulin (3 out of 4) and 87.50% β-tubulin genes (7 out of 8) showed higher expression levels in SATR than in 93-11 on the 4th day after SSAS treatment (Figure 2B). These results indicate that SATR seedlings had higher expression levels of tubulin genes than 93-11 after SSAS.

Taken together, these results indicate that the hemicellulose and pectin biosynthesis pathway and tubulin genes were more activated in SATR seedlings than in 93-11 induced by salt–alkali stress.

### 2.4. SATR Had Higher Gene Expressions in Cutin, Suberin and Wax Biosynthesis Pathway under SSAS

**Figure 3:**
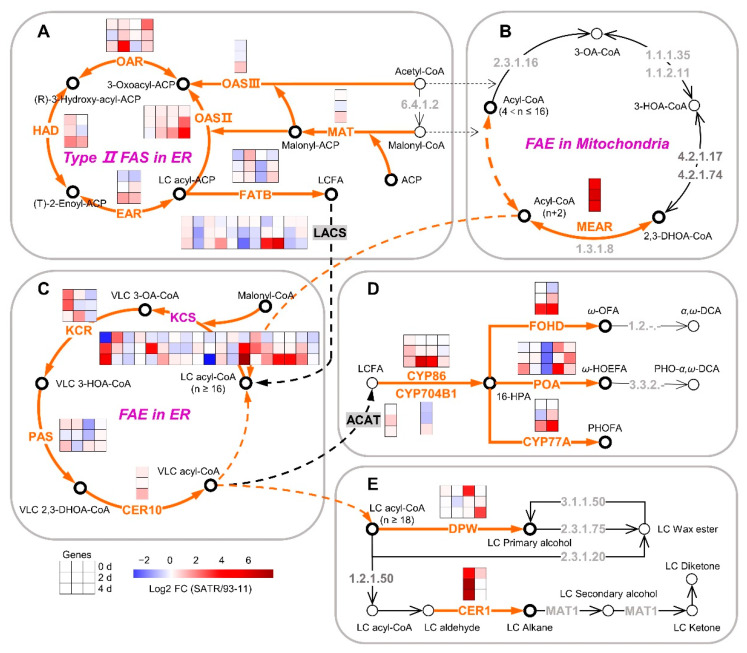
Gene expression analysis in cutin, suberin and wax biosynthesis pathway between SATR and 93-11 under SSAS. (**A**–**E**) Gene expression analysis of the cutin, suberin and wax biosynthesis pathway mainly including Type II fatty acid biosynthesis (FAS) in endoplasmic reticulum (ER) (**A**), fatty acid elongation (FAE) in mitochondria (**B**), FAE in ER (**C**), cutin and suberin (**D**) and wax biosynthesis (**E**) between SATR and 93-11 under SSAS for 0 days, 2 days and 4 days. The meanings of arrows, open circles and the black symbols beside them, orange name, and color of small inner cell in the big composite box are the same as that indicated in Figure 2. The detailed information of these enzyme-encoding genes and full names of the metabolites were summarized in Table S4, respectively. The thresholds for DEGs are |log2 [fold change (SATR/93-11)]| > 0.5850 (1.5-fold change value of gene expressions in SATR compared with 93-11) and *p*-value < 0.05.

To explore the roles of cutin, suberin and wax biosynthesis to the SSAS tolerance of SATR seedlings, we further analyzed gene expression levels between SATR and 93-11 in cutin, suberin and wax biosynthesis pathways. At least 21 groups of enzymes, encoded by 78 corresponding genes, are involved in this pathway through a series of catalytic reactions (Figure 3A–E). First of all, the Type II FAS in ER is catalyzed by seven groups of enzymes in ER, including MAT, OASII, OASIII, OAR, HAD, EAR and FATB, which are encoded by 18 genes, respectively. Approximately two-thirds of the encoding genes of these enzymes had markedly higher expression levels in SATR than 93-11 on the 4th day after SSAS treatment (Figure 3A). In addition, MEAR is the key enzyme in the FAE pathway in mitochondria, responsible for the shorter chain fatty acid (4 < *n* ≤ 16) elongation by converting the 2,3-DHOA-CoA into acyl-CoA (*n* + 2) at the last step in this pathway. The MEAR encoding gene showed a 39.92-, 69.40- and 52.05-fold higher expression level in SATR compared with 93-11 on the 0th, 2nd and 4th day after SSAS treatment, respectively (Figure 3B).

Subsequently, the LC acyl-CoA (*n* ≥ 16) in FAE pathway in ER is converted to VLC acyl-CoA (*n* ≥ 16) by the catalysis of the KCS, KCR, PAS and CER10 in sequence. Excluding non-DEGs, most DEGs (83.33%) which encode the four enzymes in this pathway also showed higher expression levels in SATR than 93-11 on the 4th day after SSAS treatment (Figure 3C).

Furthermore, the VLC acyl-CoA is finally converted to cutin and suberin such as ω-OFA, ω-HOEFA and PHOFA by six groups of enzymes. These enzymes are ACAT, CYP86, CYP704B1, FOHD, POA and CYP77A, which are encoded by 15 genes (Figure 3D). Additionally, the VLC acyl-CoA is also converted to wax such as LC primary alcohol and LC alkane by at least two groups of known enzymes, which are DWP and CER1 encoded by four and two genes, respectively (Figure 3E). Taken as a whole, excluding non-DEGs, most DEGs (90.91%) which encode the eight groups of enzymes exhibited higher expression levels in SATR than 93-11 on the 4th day after SSAS treatment (Figure 3D,E).

Collectively, these results indicate that SATR seedlings have higher gene expression levels in cutin, suberin and wax biosynthesis pathway than 93-11 under SSAS.

### 2.5. Mapping of the Candidate Genes Controlling Salt–Alkali Tolerance in SATR

**Figure 4:**
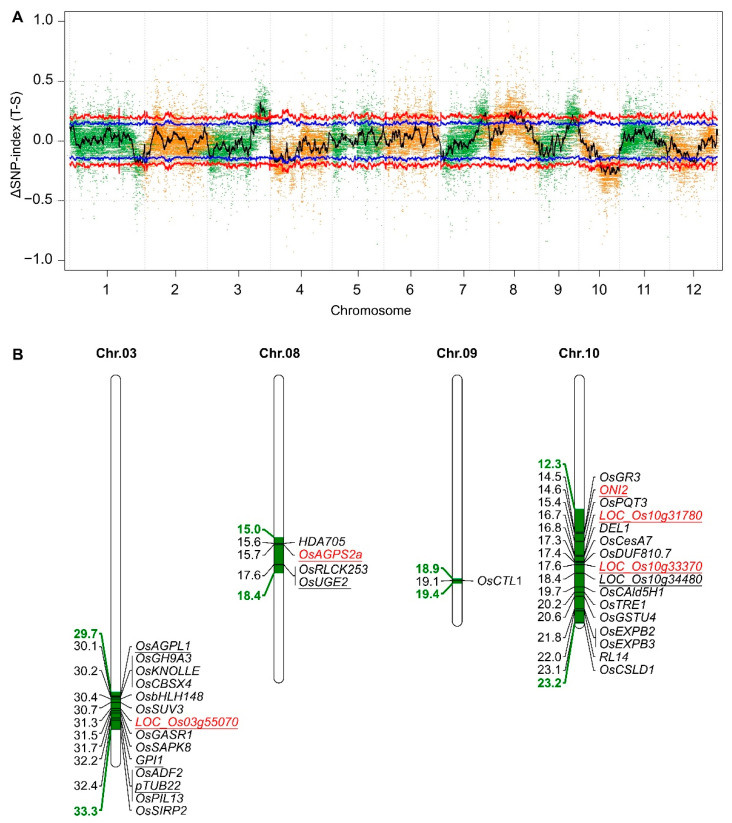
Mapping of the candidate genes of salt–alkali tolerance in SATR. (**A**) Whole-genome mapping of the candidate genes controlling saline–alkali tolerance in SATR by GBTS. Dense and green or orange spots in the plot represent the ∆SNP-index (T-S) between T-bulk and S-bulk (T-bulk–S-bulk). T-bulk and S-bulk represent the bulk DNA samples extracted from 30 individual seedlings showing the most extremely tolerant and susceptible phenotypes against SSAS treatment, respectively. Black curved line means the average ∆SNP-index (T-S) calculated based on 1 Mb interval with a 100 kb sliding window. Red and blue curved lines in the plot mark the 99% (*p* < 0.01) and 95% (*p* < 0.05) statistical confidence intervals under the null hypothesis of no gene was identified within the confidence intervals. (**B**) Locations of the predicted candidate genes controlling saline–alkali tolerance in SATR. The chromosome 7 is not shown because the predicted candidate genes controlling saline–alkali tolerance in SATR were not identified in the mapping region on chromosome 7. Green and bold numerals on the left side of the chromosomes indicate the physical positions of the preliminary mapping region in which the predicted candidate genes are located. Black numerals on the left side of the chromosomes indicate the physical positions of the corresponding predicted candidate genes on the right side of the chromosomes within the preliminary mapping regions. Underlined genes represent the encoding genes of the hemicellulose and pectin biosynthesis pathway and the β-tubulin protein (Figure 2), as well as the encoding genes of the cutin, suberin and wax biosynthesis pathway (Figure 3). Genes marked with red color mean expressions levels of these genes were significantly higher in SATR seedlings than 93-11 on the 4th day after SSAS treatment. Chr., chromosome; GBTS, genotyping by target sequencing and liquid chip.

We subsequently performed preliminarily mapping of the candidate genes controlling salt–alkali tolerance in SATR. A total of five mapping regions of the candidate genes were identified, which are located in the five regions with 3.6 (29.7–33.3), 1.9 (25.8–27.7), 3.4 (15.0–18.4), 0.5 (18.9–19.4) and 10.9 (12.3–23.2) Mbp on chromosomes 3, 7, 8, 9 and 10 above the 99% confidence intervals, respectively (Figure 4A,B). By a series of bioinformatics and literature analysis (see Section 4.9 of Materials and Methods) for the genes within the mapping regions, 9 catalyticase-encoding genes in the hemicellulose, pectin, cutin, suberin and wax biosynthesis pathways, one β-tubulin encoding gene, and the other 25 genes reported to directly control or be related to salt stress, osmotic stress or drought stress were identified (Figure 2, Figure 3 and Figure 4B; Table S5). These 35 highlighted candidate genes are considered as the potential and possible causal genes which control the salt–alkali tolerance in SATR. Among them, the five genes containing *OsAGPL1*, *OsAGPS2a*, *PGI1*, *OsUGE2* and *LOC_Os03g55070* are catalyticase genes in the hemicellulose and pectin biosynthesis pathway. The four genes containing *LOC_Os10g31780*, *ONI2*, *LOC_Os10g33370* and *LOC_Os10g34480* are catalyticase genes in the cutin, suberin and wax biosynthesis pathway. The *pTUB22* gene encodes β-tubulin protein. Furthermore, expression levels of the five genes containing *OsAGPS2a*, *LOC_Os03g55070*, *LOC_Os10g31780*, *ONI2* and *LOC_Os10g33370* were significantly higher in SATR seedlings than 93-11 on the 4th day after SSAS treatment (Figure 2, Figure 3 and Figure 4B). Therefore, these results further indicate that the cell wall biogenesis associated pathways and the inside candidate genes are vital for modulating salt–alkali tolerance in SATR seedlings. The causal genes which confer the salt–alkali tolerance in SATR need to be finely mapped and cloned in the future.

### 2.6. Validation of RNA-Seq Data through qRT-PCR Analysis

**Figure 5:**
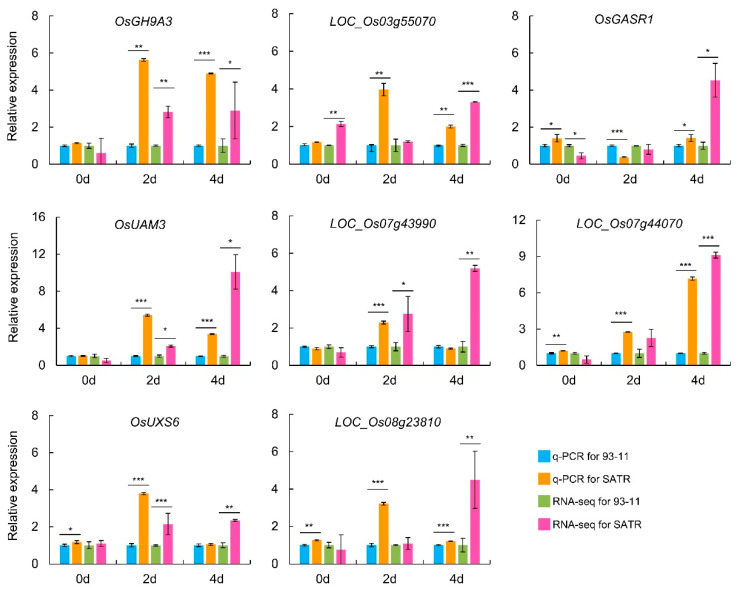
Comparison analysis of gene expression levels and patterns of the selected genes between SATR and 93-11 by qRT-PCR and RNA-seq results. The value of *OsActin* mRNA was used as the reference control for data normalization. The expression levels of the eight genes in 93-11 were scaled to 1 for qRT-PCR analysis under SSAS for 0 days, 2 days, and 4 days, respectively. The FPKM values of the eight genes in 93-11 were scaled to 1 for gene expression analysis of RNA-seq data under SSAS for 0 days, 2 days, and 4 days, respectively. FPKM, fragments per kilobase of transcript per million mapped reads. Data is given as mean ± SD, which was obtained from three independent biological replicates, and each sample contained five mixed seedlings. Student’s *t*-test, * *p* < 0.05, ** *p* < 0.01, *** *p* < 0.001.

To experimentally validate the reliability of RNA-seq results, eight genes in the hemicellulose and pectin biosynthesis pathway were selected to compare the gene expression patterns between qRT-PCR analysis and RNA-seq results in SATR and 93-11 seedlings, including *OsGH9A3*, *LOC_Os03g55070*, *OsGASR1*, *OsUAM3*, *LOC_Os07g43990*, *LOC_Os07g44070*, *OsUXS6* and *LOC_Os08g23810*. Subsequent analysis indicates that expression patterns of these genes are very similar between qRT-PCR and RNA-seq results. A total of 75.00% tested data in qRT-PCR analysis showed consistent relative expression tendency with that in RNA-seq results between SATR and 93-11 seedlings. Taking the expression data of qRT-PCR and RNA-seq analysis together, the eight genes showed higher expression levels in SATR than 93-11 on the 2nd (87.50% genes) and 4th (93.75% genes) days after SSAS treatment (Figure 5). Moreover, considering that the RNA-seq data had high-quality reads, high mapping percentages of clean reads to the reference genome and good repeatability within independent biological replicates (Tables S1 and S2; Figure S3A,B), these results collectively indicate reliability of the RNA-seq data in this study.

### 2.7. SATR Seedlings Accumulated Higher Levels of Hemicellulose and Pectin under SSAS

**Figure 6:**
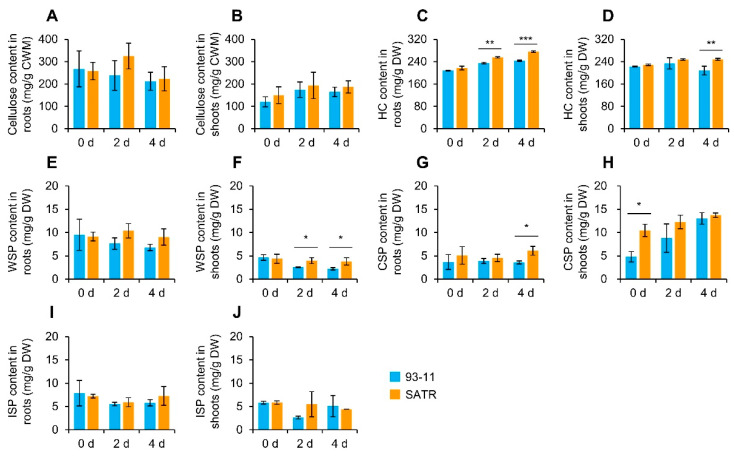
Comparisons of cellulose, hemicellulose and pectin contents between SATR and 93-11 under SSAS. Contents of cellulose in roots (**A**) and shoots (**B**), hemicellulose (HC) in roots (**C**) and shoots (**D**), water-soluble pectin (WSP) in roots (**E**) and shoots (**F**), covalently soluble pectin (CSP) in roots (**G**) and shoots (**H**), and ionic-soluble pectin (ISP) in roots (**I**) and shoots (**J**) in STAR and 93-11 seedlings under SSAS for 0 days, 2 days, and 4 days, respectively. Data is given as mean ± SD, which was obtained from three independent biological replicates, and each sample contained 13 mixed seedlings. Student’s *t*-test, * *p* < 0.05, ** *p* < 0.01, *** *p* < 0.001.

We further measured contents of the main cell wall polysaccharides between SATR and 93-11 in shoots and roots under SSAS for 0 days, 2 days and 4 days. There was no significant difference in cellulose contents in both roots and shoots between SATR and 93-11 seedlings on the 0th, 2nd and 4th day after SSAS treatment (Figure 6A,B). Nevertheless, both of them exhibited continually increasing hemicellulose contents after SSAS, but SATR accumulated significantly higher levels of hemicellulose in the roots than 93-11 on the 2nd and 4th day after SSAS treatment (Figure 6C). Besides, SATR shoots not only displayed continually increased hemicellulose content after SSAS, but also accumulated significantly higher levels of hemicellulose in shoots than 93-11 on the 4th day after SSAS treatment (Figure 6D). Moreover, although there was no significant difference in water-soluble pectin (WSP) contents in roots between SATR and 93-11 on the 0th, 2nd and 4th day, SATR shoots had significantly higher WSP contents in shoots on the 2nd and 4th day after SSAS treatment, which are 35.07% and 41.79% higher increases than that of 93-11, respectively (Figure 6E,F). Furthermore, both SATR and 93-11 showed continually increasing covalently soluble pectin (CSP) contents in shoots after SSAS. However, SATR had significantly much higher CSP contents than 93-11 in both of the roots under SSAS for 4 days and the shoots under SSAS for 0 days, indicated by 70.44% and 117.70% increases, respectively (Figure 6G,H). In addition, there was no significant difference in ionic-soluble pectin (ISP) contents between SATR and 93-11 in both of the roots and shoots on the 0th, 2nd and 4th day after SSAS treatment (Figure 6I,J).

In summary, these results indicate that SATR seedlings accumulated higher levels of hemicellulose and pectin than 93-11 under SSAS, which can be attributed to the higher expression levels of genes in the hemicellulose and pectin biosynthesis pathway, and thereby modulating SSAS tolerance in SATR seedlings. It further indicates that the key genes in the hemicellulose and pectin biosynthesis pathway may play vital roles in conferring salt–alkali tolerance in SATR.

### 2.8. The Hemicellulose Biosynthesis Gene OsCSLD4 Confers Salt–Alkali Stress Tolerance in Rice

**Figure 7:**
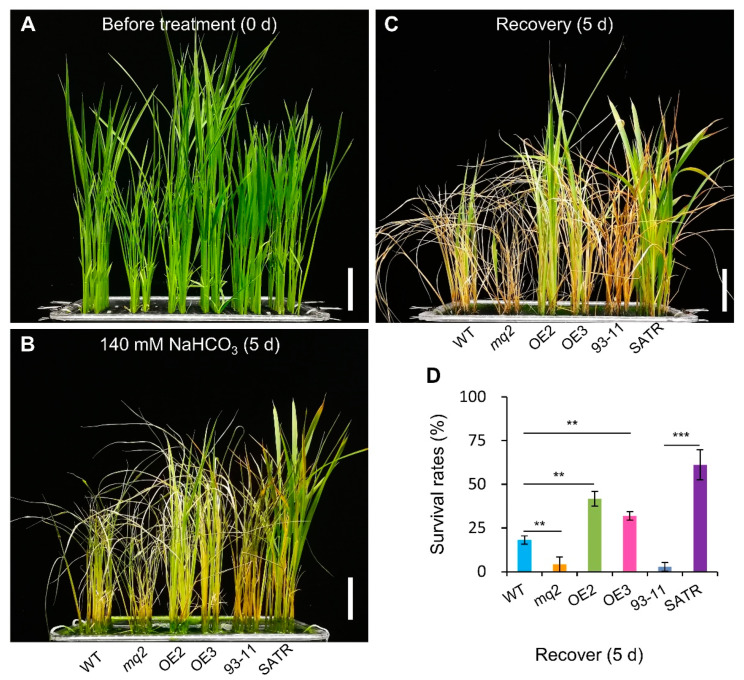
Saline–alkali tolerance analysis of *OsCSLD4* function-disrupted mutant and overexpression lines. (**A**–**C**) Performance of *OsCSLD4* function-disrupted mutant (*mq2*), *OsCSLD4*-overexpression lines (OE2 and OE3) compared with their wild-type plants (Kitaake, WT), as well as 93-11 and SATR seedlings before SSAS treatment (**A**), on the 5th day after SSAS treatment (**B**), and after 5 days of recovery without SSAS treatment (**C**), respectively. (**D**) Survival rate of WT, *mq2*, OE2, OE3, 93-11 and SATR seedlings after 5 days of recovery. Bar = 5 cm (**A**–**C**). Data is given as mean ± SD, which was obtained from three independent biological replicates, and each sample contained 24 seedlings. Student’s *t*-test, ** *p* < 0.01, *** *p* < 0.001.

We further selected the key 1,4-β-D-xylan synthase gene (*OsCSLD4*) in the hemicellulose biosynthesis pathway, and analyzed the salt–alkali tolerance of its function-disrupted mutant (*mq2*) and overexpression lines (OE2 and OE3). Kitaake (wild type), *mq2* mutant, OE2, OE3, SATR and 93-11 seedlings grew healthily with vivid green leaves before SSAS, although the *mq2* seedlings were more stunted than the wild type due to functional disruption of *OsCSLD4* gene (Figure 7A). However, the *mq2* seedlings were severely withered after 5 days of SSAS and not able to grow and survive from 5 days of recovery after SSAS, indicated by the extremely low survival rate of 4.17% compared with the wild type with a survival rate of 18.06%. In contrast, both of OE2 and OE3 *OsCSLD4*-overexpression seedlings were more tolerant to SSAS than wild-type seedlings with greener but less withered leaves after SSAS for 5 days. The survival rates of OE2 and OE3 seedlings were higher than that of the wild type by 23.61% and 13.89%, respectively. In addition, 93-11 seedlings displayed the most susceptible phenotype similar to the *mq2* mutant under SSAS. On the contrary, SATR seedlings exhibited the strongest SSAS tolerance in the six rice accessions with the most green but fewest withered leaves, which was further verified by the highest survival rate of 61.11% (Figure 7B–D). Thus, functional disruption of *OsCSLD4* gene decreased the salt–alkali tolerance, but overexpression of *OsCSLD4* increased the salt–alkali tolerance of rice seedlings, which indicates that *OsCSLD4* confers salt–alkali stress tolerance in rice.

### 2.9. OsCSLD4 Positively Regulates Grain Yield under Salt–Alkali Stress in Rice

**Figure 8:**
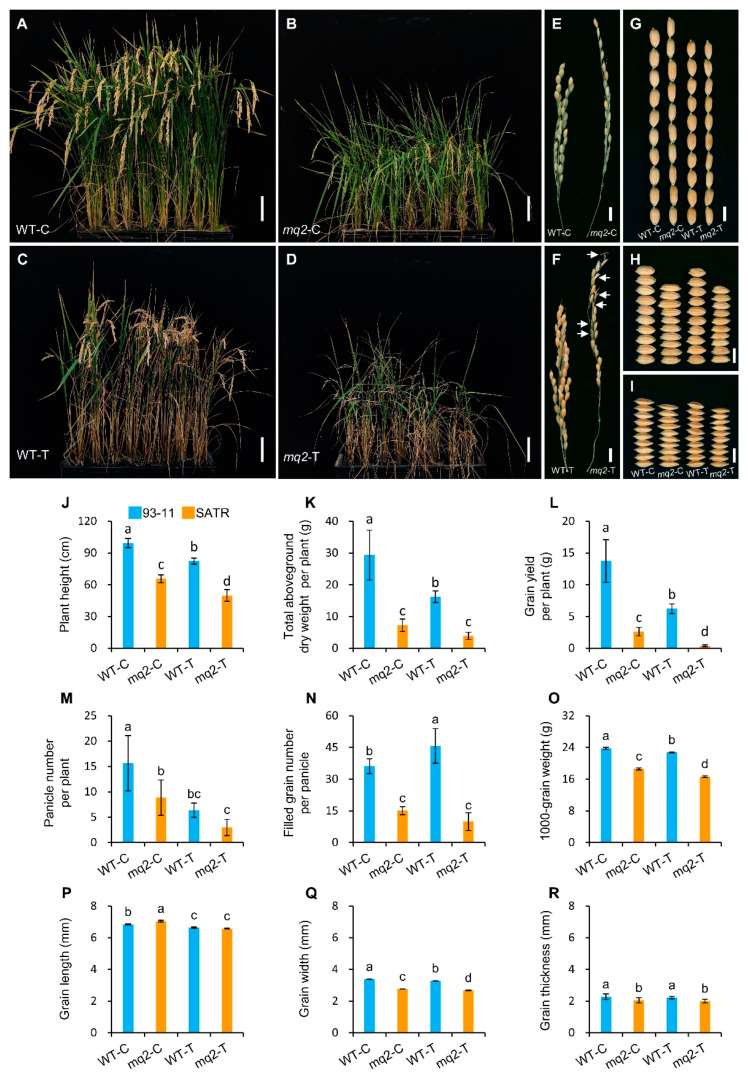
Effects of *OsCSLD4* on the biomass and grain yield under NaHCO_3_ treatment. (**A**,**B**) Morphologies of wild-type Kitaake (WT-C) (**A**) and *mq2* mutant (*mq2*-C) plants (**B**) at maturation stage which were grown in a same cultivating box under controlled growth conditions without NaHCO_3_ treatment. (**C**,**D**) Morphologies of wild-type (WT-T) (**C**) and *mq2* (*mq2*-T) (**D**) plants at maturation stage which were grown in a same cultivating box and treated with approximately 8‰ NaHCO_3_ in the soil. (**E**,**F**) Panicle morphologies of WT-C and *mq2*-C plants (**E**) as well as WT-T and *mq2*-T plants (**F**). (**G**–**I**) Grain length (**G**), grain width (**H**) and grain thickness (**I**) phenotypes of WT-C, *mq2*-C, WT-T and *mq2*-T plants. (**J**–**R**) Statistics of plant height (**J**), total aboveground dry weight per plant (**K**), grain yield per plant (**L**), panicle number per plant (**M**), filled grain number per panicle (**N**), 1000-grain weight (**O**), grain length (**P**), grain width (**Q**) and grain thickness (**R**) of WT-C, *mq2*-C, WT-T and *mq2*-T plants. WT, the wild-type plants (Kitaake) harboring the functional *OsCSLD4* gene; *mq2*, the *OsCSLD4* function-disrupted mutant of Kitaake. The blue and orange bars indicate wild-type and *mq2* mutant, respectively (**J**–**R**). Bar = 10 cm (**A**–**D**), 10 mm (**E**,**F**) and 5 mm (**G**–**I**), respectively. Data is given as mean ± SD, which was obtained from eight independent biological replicates (**J**–**N**) or three independent biological replicates (**O**–**R**). Each independent replicate contained one plant (**J**–**N**), or ≥ 200 grains (**O**–**Q**), or 40 grains (**R**). The different letter on the bar in each bar chart (**J**–**R**) indicates the significant difference at *p* < 0.05 level obtained by ANOVA with Duncan’s new multiple range test.

Subsequently, the wild-type and *mq2* plants were treated with NaHCO_3_ from the early tillering stage to further analyze the function of *OsCSLD4* in regulating biomass and grain yield under salt–alkali stress. Without NaHCO_3_ treatment, the *mq2* plants showed obviously decreased aboveground biomass, grain yield as well as slender panicles and grains compared with wild plants (Figure 8A,B,E,G,K,L,P–R; Table S7), which indicates that the *OsCSLD4* gene has essential roles in controlling biomass and grain yield in wild-type plants in normal growth conditions. On the other hand, both wild-type and *mq2* plants displayed premature leaf senescence by NaHCO_3_ treatment (Figure 8C,D). Moreover, *mq2* plants also exhibited significantly and largely reduced aboveground biomass and grain yield by 76.06% and 92.30% compared with the wild type under salt–alkali treatment, which resulted from simultaneously reduced plant height, panicle number per plant, filled grain number per panicle and 1000-grain weight by 39.20%, 52.94%, 78.34% and 26.68%, respectively (Figure 8J–O; Table S7). The *mq2* plants exhibited slender panicles and grains than wild-type plants indicated by increased panicle length, grain length/width ratio and grain length/thickness ratio by 5.23%, 21.54% and 9.81%, respectively (Figure 8F–I,P–R; Table S7). In addition, spikelet degeneration occurred in the panicles of *mq2* plants by salt–alkali treatment which consequently caused the reduced total grain number per panicle and affected the grain yield (Figure 8F; Table S7).

The decreasing percentage of grain yield of *mq2* mutant with the *OsCSLD4* function-disrupted gene relative to the wild type under salt–alkali stress was 7.85% higher than the decreasing percentage without salt–alkali treatment (Figure 8L; Table S7). Thereinto, the decreasing percentages of panicle number per plant, filled grain number per panicle and 1000-grain weight of *mq2* mutant relative to the wild type under salt–alkali stress were 9.74%, 20.12% and 4.91% higher than the decreasing percentages without salt–alkali treatment, respectively (Figure 8M–O; Table S7). These results indicate that the *OsCSLD4* gene not only plays essential roles in controlling biomass and grain yield under normal growth conditions, but also plays indispensable roles in positively regulating grain yield by resisting the loss of biomass and grain yield under salt–alkali stress in rice.

## 3. Discussion

In this study, we revealed that induced higher expression levels of cell wall matrix polysaccharide biosynthesis pathway genes including *OsCSLD4* and correspondingly higher accumulation levels of hemicellulose and pectin, as well as adaptive physio-biochemical and phenotypic responses modulate salt–alkali tolerance in SATR seedlings. However, the detailed biological processes and underlying molecular mechanisms that how SATR seedlings respond and adapt to salt–alkali stress are still under the tip of the iceberg, which are further discussed and summarized as follows.

**Figure 9:**
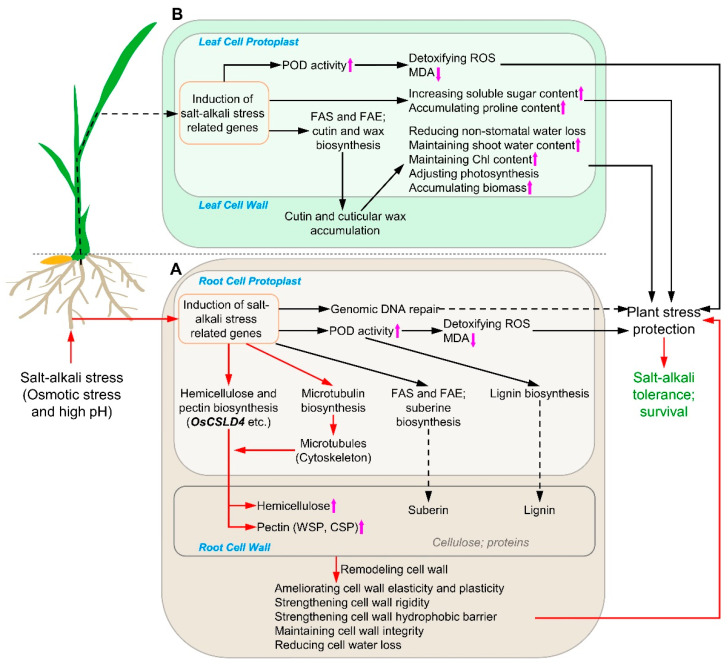
A proposed model depicting how SATR adapts to salt–alkali stress. (**A**,**B**) The metabolism pathways, regulatory networks and molecular mechanisms depicting how SATR seedlings adapt to salt–alkali stress in roots (**A**) and shoots (**B**), respectively. Solid arrow and dashed arrow indicate sequential step (or process) and predicted sequential step (or process) in the metabolism pathways and regulatory networks, respectively. The metabolism pathways and regulatory networks along with the red arrows are the most key pathways and regulatory networks which modulate salt–alkali tolerance of SATR. Purple up or down arrow indicates corresponding component content is significantly higher or lower in SATR seedlings than 93-11 after SSAS, respectively. Chl, chlorophyll; WSP, water-soluble pectin; CSP, covalently soluble pectin; FAS, Type II fatty acid biosynthesis; FAE, fatty acid elongation; MDA, malondialdehyde; POD, peroxidase; ROS, reactive oxygen species.

After being subjected to salt–alkali stress, the signal of combined salt–alkali stress including osmotic and high pH stress is perceived by the root cells in SATR seedlings (Figure 9A). Our results showed that there were not significant differences in the contents of major cell wall components including cellulose, hemicellulose, WSP, ISP and CSP between SATR and 93-11 roots before salt–alkali stress (Figure 6), which indicates that their roots had similar physio-biochemical conditions in major cell wall components before salt–alkali stress.

After the salt–alkali stress signal is transduced into root cell protoplast, expressions of some salt–alkali stress-related genes are activated (Figure 9A). For example, expressions of DNA repair genes are activated in the nucleus to repair the DNA damage resulted from salt–alkali stress. This DNA repair mechanism is supported by our result that the nucleotide base and excision repair pathways were significantly enriched from the upregulated DEGs between SATR and 93-11 on the 2nd day after SSAS treatment (Figure S8A), which suggests that SATR seedlings may have stronger ability to repair the DNA damage in root cells to cope with salt–alkali stress than 93-11. Besides, some physio-biochemical processes could be activated to deal with oxidative stress in the root cells triggered by salt–alkali stress. For instance, POD is a key antioxidase involved in detoxification of ROS [40]. SATR seedlings had higher POD activity on the 0th, 2nd and 4th days after SSAS treatment but lower MDA content than 93-11 on the 4th day after SSAS treatment (Figure 1L,M), which suggests that SATR has stronger ROS scavenging ability to reduce the oxidative stress in the root cells. This deduction is also supported by the result that the oxidation–reduction process is the most significantly enriched GO BP (GO biological processes functional category) term from the downregulated DEGs on the 2nd and 4th day after SSAS treatment (Figures S6D and S7D). Thus, the DNA-repairing and oxidative stress-reducing mechanisms could protect the SATR seedlings against the salt–alkali stress in the root cells.

On the other hand, POD is a multifunctional key enzyme which also catalyzes the last reaction step of the lignin biosynthesis (KEGG, https://www.kegg.jp/kegg-bin/show_pathway?dosa00940) (accessed on 20 October 2022). Thus, higher POD activity and gene expression levels in suberin, hemicellulose and pectin biosynthesis pathways (Figure 1M, Figure 2A and Figure 3D) could increase the biosynthesis of lignin, suberin, hemicellulose and pectin in SATR root cells under SSAS. In addition, the cytoskeletons including microtubule are not only involved in cellulose microfibril biosynthesis [41], but also directly involved in vesicle vacuolar trafficking [42,43]. After biosynthesizing in the Golgi apparatus [44], the non-cellulosic cell wall polysaccharides are further transported and secreted to apoplast by Golgi-derived vesicle trafficking, and associated with newly synthesized cellulose microfibrils in the cell wall [45]. Our results showed not only remarkably higher expression levels of tubulin genes, but also higher accumulation levels of hemicellulose and CSP in SATR roots than 93-11 under SSAS (Figure 2B and Figure 6C,G). It indicates that more hemicellulose and pectin as well as possibly more lignin and suberin are transported and deposited to the cell wall of root cells under salt–alkali stress. Furthermore, the *nd1* function-disrupted mutant of *OsCSLD4* gene in the hemicellulose biosynthesis pathway showed not only reduced xylan and cellulose contents in culms [38], but also decreased salt tolerance compared with its wild-type plants [39], which is in line with our results.

Cellulose, hemicellulose, pectin, suberin and lignin are major components in the cell wall of rice roots. Thereinto, the hemicellulose and CSP could form link with each other through covalent bonds, and further strengthen the elasticity and plasticity of cell wall [23,24,25,46]. The lignin and suberin, forming important hydrophobic barriers in the cell wall, could further strengthen the mechanical property of cell wall to prevent water loss [26,30,47]. Thus, deposition and accumulation of more hemicellulose and pectin as well as possibly more lignin and suberin in the cell wall of SATR root cells can remodel the cell wall, strengthen cell wall mechanical properties and barrier, and maintain cell wall integrity, which helps the SATR seedlings to adapt to the salt–alkali stress in the roots.

After subjected to salt–alkali stress, it not only causes osmotic stress and inhibits water intake in the roots of SATR seedlings, but also subsequently transduces the stress signal into shoots through vascular system and results in physiological drought and oxidative stress accordingly. Similarly to the mechanism in roots, expressions of some salt–alkali stress-related genes and correspondingly physio-biochemical processes in SATR leaf cell protoplast are activated to respond to salt–alkali stress (Figure 9B). For example, ROS scavenging mechanism could also be activated to reduce oxidative stress, which is supported by the higher POD activity and lower MDA content in SATR seedlings than 93-11 under SSAS (Figure 1L,M). On the other hand, the leaf cells could decrease their osmosis potential to resist physiological drought by increasing the endocellular soluble sugar contents to protect leaf cells from the stomatal stress, which is supported by the significantly higher total soluble sugar content in SATR seedlings than 93-11 on the 2nd and 4th day after SSAS treatment (Figure 1J,K and Figure 6F). Although SATR seedlings had lower proline contents than 93-11 on the 0th and 2nd day after SSAS treatment, both of them showed remarkably and continuously increased proline contents after SSAS (Figure 1J). These adaptive physio-biochemical mechanisms can protect the cells in SATR shoots against the stomatal and oxidative stresses caused by salt–alkali stress.

On the other hand, higher gene expressions in cutin and suberin as well as wax biosynthesis pathway in SATR seedlings than 93-11 under SSAS (Figure 3D,E) may increase the content and deposition of cutin and cuticular waxes on the leaf cell walls. More cuticular waxes deposition and accumulation can increase cell wall cutinization levels, hence helping plant leaves to reduce non-stomatal water loss [48,49]. Correspondingly, SATR seedlings can benefit from higher shoot water content to maintain higher chlorophyll content (Figure 1H,O) and adjust photosynthesis rate in the leaves, which helps the SATR seedlings to maintain continuous growth and accumulate more biomass than 93-11 under salt–alkali stress (Figure 1A–G,M and Figure S3A–D). It finally helps the SATR seedlings to adapt to salt–alkali stress and survive.

## 4. Materials and Methods

### 4.1. Establishment of a Large-Scale Screening System for Obtaining Salt–Alkali-Tolerant Rice Germplasms

We constructed a large-scale screening system with rice seedlings by 140 mM Na-HCO_3_ (pH 9.20) treatment [11,12,13,14,15,16,17,18] in a greenhouse. The large-scale screening system was constructed by modifying the method of Bado et al. (2020) [50] (Figure S1A–D). Rice seeds were surface-sterilized with 1% NaClO, soaked in an incubator, at 35 °C, for 2 days, and then germinated, at 30 °C, in the greenhouse. Each of the 33 black 96-well PCR plates with the bases cut off were fixed together before transplanting of germinated seedlings, and placed into a large black cultivating box (670 × 405 × 160 mm). After germination, healthy and uniform seedlings at one-leaf stage were transferred to these 96-well PCR plates. A total of 2376 seedlings of 99 rice accessions were transplanted into the 33 fixed and combined 96-well PCR plates (24 seedlings for each rice accession, and 72 seedlings of every 3 rice accessions in each 96-well PCR plate). Subsequently, the combined 33 PCR plates with 2376 seedlings were transferred to the cultivating box and cultured with Yoshida’s rice nutrient solution (pH 5.5) (Figure S1) [51].

These seedlings were cultured in the greenhouse with a light/dark cycle of 14/10 h at 29/21 °C and a relative humidity of around 70%. The light intensity was 750–850 μmol m^−2^ s^−1^. Seedlings at 2–3 leaf stage were treated with the nutrient solution containing 140 mM NaHCO_3_ (simulating SSAS; pH 9.20) for 7 days. Subsequently, the seedlings were recovered for 5 days in new nutrient solution without NaHCO_3_. The tolerance of rice seedlings to SSAS is evaluated mainly by the visual phenotypes of leaf color, green leaf number, extent of leaf rolling and wilting, and especially the survival rate. A seedling with completely wilted leaves and not being able to grow and survive after 5 days of recovery is judged as a dead seedling.

### 4.2. Plant Materials

The SATR is a SSAS-tolerant germplasm of tropical *japonica* rice, which was screened from more than 700 rice accessions in our lab using the large-scale screening system. 93-11 is a popular rice cultivar and it is cultivated widely in China [52], which was used as a susceptible control for SATR against SSAS in this study. The function-disrupted mutant (*mq2*) and the other two overexpressing lines (OE2 and OE3) of the *OsCSLD4* gene were all generated from the Kitaake genetic background (wild type) and kindly provided by Dr. Zhijin Zhang (Biotechnology Research Institute, Chinese Academy of Agricultural Sciences, Beijing 100081, China) [39].

### 4.3. Identification and Evaluation of the SSAS Tolerance between SATR and 93-11

The growth conditions and treatment of SATR and 93-11 seedlings are the same as that in the large-scale screening system, except that the seedlings were cultured in a smaller cultivating box with Yoshida’s rice nutrient solution and subsequently treated with SSAS, salt or alkali stress. Each culture box contained 24 seedlings for SATR and 93-11, respectively. Specifically, seedlings at 2–3 leaf stage were treated with nutrient solution containing 140 mM NaHCO_3_ (SSAS, pH 9.20), 140 mM NaCl (salt stress, pH 5.50) or Na_2_CO_3_ (alkali stress, pH 10.20) (1, 2, 3, 4 and 5 days for SSAS treatment, 9 days for salt stress or alkali stress treatment) and recovered for subsequently another 5 days, respectively. The tolerance of rice seedlings against SSAS, salt stress or alkali stress is evaluated and judged by the same standards described in the large-scale screening system.

### 4.4. Identification and Evaluation of the SSAS Tolerance of OsCSLD4 Function-Disrupted Mutant and Overexpressing Lines

For analyzing the SSAS tolerance of the *OsCSLD4* function-disrupted mutant and overexpressing lines, seedlings were cultured, treated and identified using the same conditions as in the above large-scale screening system, except using smaller cultivating boxes. For analyzing the function of *OsCSLD4* in regulating biomass and grain yield under salt–alkali stress, the Kitaake and *mq2* seedlings were grown in a same big cultivating box with field soil. The control group was not treated while the treated group was treated with 80 mM NaHCO_3_ nutrient solution beginning from the early tillering stage; in addition, the NaHCO_3_ salinity in the soil of the treated group was up to approximately 8‰ at the end of tilling stage. The other growth conditions such as temperature, illumination and fertilization in a greenhouse were the same as in the above large-scale screening system.

### 4.5. Measurement of Phenotypic Parameters

The seedling height and primary root length of fresh seedlings were measured with a steel scale ruler, and the shoot diameter was measured with an electronic vernier caliper. Fresh seedlings were weighed and then dried in an oven, at 100 °C, to constant weight. The dry weight and water content were further weighed and calculated, respectively. Three independent biological replicates were sampled for the measurement of these indexes. Each independent biological replicate contained 5 mixed fresh shoots excepting 24 seedlings per independent biological replicate used for the statistics of survival rate.

### 4.6. Measurement of Physio-Biochemical Parameters

The shoots of fresh seedlings were used to measure chlorophyll content, malondialdehyde content, POD activity and SOD activity on the 0th, 1st, 2nd and 4th day after SSAS treatment. The shoots on the 0th, 1st, 2nd and 4th day after SSAS treatment and after 4 days of recovery were dried, at 100 °C, to constant weight for the determination of proline and total soluble sugar contents.

#### 4.6.1. Measurement of Total Chlorophyll Content

The fresh shoots were sampled and ground into fine powders with liquid nitrogen quickly. Approximately 0.10 g powders were collected, weighed and extracted with 5 mL 80% (*v*/*v*) cold acetone in the dark for at least 24 h until the tissues became white completely. Then, the samples were mixed and centrifuged for 10 min. The absorbances of the supernatants at 645 and 663 nm were determined by a Microplate Reader (Thermo Scientific™ Varioskan™ LUX, Thermo Fisher Scientific, Inc., Waltham, MA, USA). Total chlorophyll content (mg g^−1^ FW) = (20.29 × *A*_645_ + 8.02 × *A*_663_) × *v*/*w*, where *A*_645_ and *A*_663_ indicate the absorbance at 645 and 663 nm, respectively [53]. Three independent biological replicates were sampled for this experiment, and each sample contained 5 mixed fresh shoots.

#### 4.6.2. Measurement of Malondialdehyde Content

The fresh shoots were harvested, weighed, and ground into fine powders quickly with liquid nitrogen, homogenized with 1 mL 10% (*w*/*v*) trichloroacetic acid, then centrifuged at 4 °C and 1500 *g* for 10 min. The supernatant (1 mL) was added into a centrifuge tube and mixed with 1 mL 0.67% (*w*/*v*) thiobarbituric acid. Subsequently, the mixture was heated in boiling water for 15 min, then cooled to room temperature in ice water immediately and centrifuged at 4 °C and 2350 *g* for 10 min. The absorbances (*A*_450_, *A*_532_ and *A*_600_) of supernatant were determined (using water as control). MDA content (nmol/g FW) = [6.452 × (*A*_532_ − *A*_600_) − 0.56 × *A*_450_] × V_T_/(V_0_ × W), where *A*_450,_ *A*_532_ and *A*_600_ indicate the absorbances at 450, 532 and 600 nm, respectively [54]. Three independent biological replicates were sampled for this experiment, and each sample contained 5 mixed fresh shoots.

#### 4.6.3. Determination of POD and SOD Activity

The fresh shoots were harvested, ground into fine powders quickly with liquid nitrogen. POD activity of fresh seedlings was determined using the method [55,56]. The SOD activity was determined with nitro blue tetrazolium as described by Fridovich et al. (1971) [57]. Three independent biological replicates were sampled for these experiments, and each sample contained 5 mixed fresh shoots.

#### 4.6.4. Measurement of Proline and Total Soluble Sugar Content

The dried shoots were collected and ground into fine powders with liquid nitrogen. The proline and total soluble sugar of shoots were extracted and determined using the ‘Proline Content Assay Kit’ and ‘Soluble Sugar Content Assay Kit’ (AKAM003C and AKSU012C, Beijing Boxbio Science and Technology Co., Ltd., Beijing, China), respectively. Three independent biological replicates were sampled for this experiment, and each sample contained 5 mixed shoots.

### 4.7. RNA-Sequencing (RNA-Seq) and qRT-PCR

#### 4.7.1. Analysis of RNA-Seq and Differentially Expressed Genes (DEGs)

The seedlings of SATR and 93-11 on the 0th, 2nd and 4th day after SSAS treatment were sampled, immediately frozen in liquid nitrogen, and stored at −80 °C for the RNA-seq and qRT-PCR analysis. The collected and stored samples were frozen in drikold and shipped to Basebio Biotechnology Co., Ltd. (Chengdu 610041, China) for RNA isolation, sample quality testing, cDNA library construction, RNA-seq and primary analysis. Three independent biological replicates were sampled for these experiments, and each sample contained 10 mixed seedlings for RNA-seq and 5 mixed seedlings for qRT-PCR, respectively.

The quality of sequencing reads was evaluated using FastQC tool (https://www.bioinformatics.babraham.ac.uk/projects/fastqc/) (accessed on 20 October 2022) and subsequent accurate alignment of reads was conducted using TopHat2 tool [58]. The rice reference genome was available in MSU database (http://rice.uga.edu/) (accessed on 20 October 2022). The alignment quality of reads was checked using RSeQC package [59], and the gene expression levels were quantitated via HTSeq-count tool [60]. In standardized analysis, the effective transcript length of each reference gene was derived from Ensembl database (http://plants.ensembl.org/index.html) (accessed on 20 October 2022). The fragments per kilobase of transcript per million fragments mapped (FPKM) value was calculated in R software and represents the gene expression level [61]. The upregulated or downregulated DEGs were identified with the criteria of |log2 [fold change (SATR/93-11)]| > 0.5850 (1.5-fold change value of gene expressions in SATR compared with 93-11) and corrected *p*-value (adjusted-*p*) < 0.05 using DESeq2 R package (1.20.0) [62]. Gene Ontology (GO) and KEGG annotations are available in the GO database (http://geneontology.org/) (accessed on 20 October 2022) and KEGG database (https://www.kegg.jp/) (accessed on 20 October 2022), respectively. GO and KEGG enrichment analysis was performed using clusterProfiler R package [63].

#### 4.7.2. RNA Isolation and qRT-PCR Assay

Total RNA was extracted using the ‘Plant Total RNA Isolation Kit Plus’ kit (Cat.No.RE-05021, FOREGENE CO., LTD., Chengdu, China). The cDNA strand was synthesized using the ‘TransScript^®^ All-in-One First-Strand cDNA Synthesis SuperMix for qPCR (One-Step gDNA Removal)’ kit (AT341-02, TransGen Biotech Co., Ltd., Beijing, China). Eight DEGs were selected for qRT-PCR analysis and the corresponding primers used in this study were listed in Table S6. The qRT-PCR was performed using the ‘PerfectStart^TM^ Green qPCR SuperMix’ kit (AQ601-02, TransGen Biotech Co., Ltd., Beijing, China). Relative expression levels of genes were calculated using the 2^−ΔΔCT^ method [64]. Three independent biological replicates were sampled for this experiment, and each sample contained five mixed seedlings.

### 4.8. Measurement of Cellulose, Hemicellulose and Pectin (WSP, CSP, ISP) Contents

The roots and shoots of SATR and 93-11 on the 0th, 2nd and 4th days after SSAS treatment were collected, ground into fine powders with liquid nitrogen, and dried in an oven, at 100 °C, to a constant weight, respectively. The dried simples were weighed and further used for measuring the content of cellulose, hemicellulose, WSP, ISP and CSP using the corresponding kits (AKSU007C, AKSU008C, AKSU012C, AKSU014C and AKSU013C, respectively; Beijing Boxbio Science and Technology Co., Ltd., Beijing, China) according to the respective operation manual. Three independent biological replicates were sampled for this experiment, and each sample contained the mixed shoots or roots of 13 seedlings.

### 4.9. Mapping of the Salt–Alkali Tolerance Genes by GBTS

For mapping the candidate genes controlling salt–alkali tolerance in SATR, we adopted a genotyping by target sequencing and liquid chip (GBTS) method [65]. We initially constructed an F_2_ segregating population by a cross between SATR (maternal plant) and 93-11 (paternal plant). Then, the 665 most tolerant seedlings and the 835 most susceptible seedlings were screened, identified, and harvested from the F_2_ segregating population containing 22,250 seedlings against SSAS treatment. Subsequently, the maternal bulk DNA (SATR-bulk) and paternal bulk DNA (93-11-bulk) of the parental plants were prepared by extracting and mixing equal amount of DNA samples from 10 individual seedlings of SATR and 93-11, respectively. In addition, DNA samples were extracted, respectively, from 30 individual seedlings from the 665 most tolerant seedlings (T-bulk) and another 30 individual seedlings from the 835 most susceptible seedlings (S-bulk) of the F_2_ segregating population; the two groups of DNA samples were mixed, respectively, with an equal amount of the 30 DNA samples in each group, finally to be served as the T-bulk and S-bulk, respectively. Then, the DNA-bulk samples frozen by drikold were shipped to MolBreeding Biotechnology Co., Ltd. (Shijiazhuang 050035, China) for GBTS using a GenoBaits Rice 40K Chip. The similar detailed protocols and methods for GBTS including DNA library construction, target sequencing and liquid chip, quality control of the sequencing data and initial data analysis are described in the references [66,67,68].

For SNP-calling, unique SNP sites were filtered in both maternal bulk (SATR-bulk) and paternal bulk (93-11-bulk) by comparing the two bulks. The intersections among unique SNP sites from the two maternal and paternal bulks and SNP sites of the progeny bulks (T-bulk and S-bulk) were taken. Then, the SNP-indexes of the screened SNP sites were calculated. Sliding window analysis was subsequently applied to calculate the SNP-index of T-bulk and S-bulk, and to calculate the ∆SNP-index between T-bulk and S-bulk (T-bulk–S-bulk) based on 1 Mb interval with a 100 kb sliding window. The ∆SNP-index (T-S) plot as well as the corresponding linkage maps were simultaneously drawn and constructed. For the ∆SNP-index (T-S) plot, the 99% (*p* < 0.01) and 95% (*p* < 0.05) statistical confidence intervals were set under the null hypothesis of no causal gene identified within the confidence intervals. Thus, the candidate genes controlling salt–alkali tolerance were judged within the regions above the 99% (*p* < 0.01) confidence intervals. To further screen candidate genes controlling salt–alkali tolerance in SATR, the gene set within the five mapping regions was intersected with the union of the four gene sets of cell wall biogenesis, salt stress, osmotic stress and drought stress.

### 4.10. Analysis of Major Agronomic Traits

Eight independent plants of Kitaake and *mq2* mutant in controlled growth conditions or treated with NaHCO_3_ were harvested and analyzed for the major agronomic traits at maturation stage according to the methods reported by Li et al. (2020) [69]. Eight independent biological replicates were performed for the measurement of the indexes of plant height, total aboveground dry weight per plant, grain yield per plant, panicle number per plant, filled grain number per panicle, panicle length, primary branch number per panicle, second branch number per panicle, total grain number per panicle, seed setting rate, and each sample contained a plant. The measurement for 1000-grain weight, grain length, grain width and grain thickness was performed in three independent biological replicates. For 1000-grain weight, grain length and grain width, each independent biological replicate contained ≥200 grains. For grain thickness, each independent biological replicate contained 40 grains.

## Data Availability

The RNA-seq raw data had been deposited in the NCBI BioProject with the accession number of PRJNA904078.

## Supplementary Materials

The following supporting information can be downloaded at: https://www.mdpi.com/article/10.3390/ijms232315019/s1. References are cited in [70,71,72,73,74,75,76,77,78,79,80,81,82,83,84,85,86,87,88,89,90,91,92].
