# Supplementary material for: Cell Wall Matrix Polysaccharides Contribute to Salt–Alkali Tolerance in Rice
Source: Int J Mol Sci. 2022 Nov 30;23(23):15019. doi: 10.3390/ijms232315019 (PMC9735747; doi:10.3390/ijms232315019)
Supplement: Supplementary file 1 [file ijms-23-15019-s001.zip › 2-1 Supplementary Figures and Tables.pdf]

International Journal of *Molecular Sciences*

Supporting Information

Cell Wall Matrix Polysaccharides Contribute to Salt–Alkali Tolerance in Rice

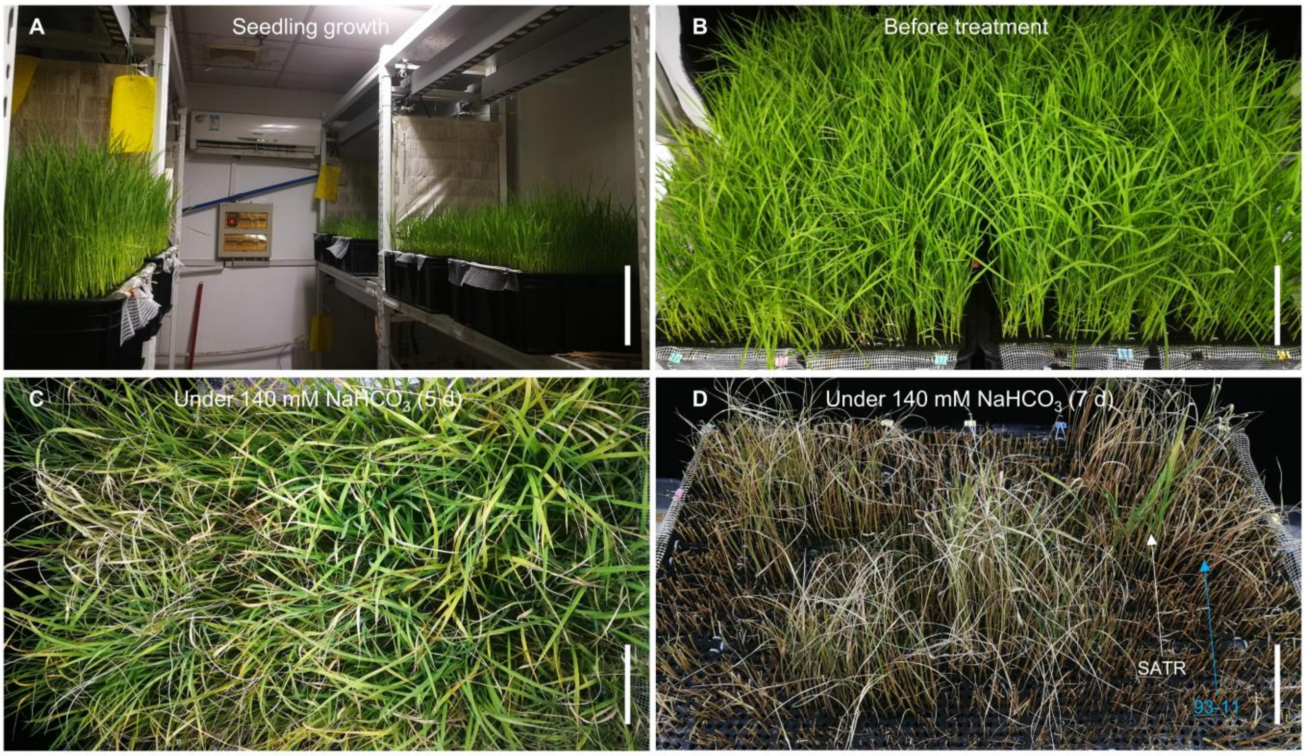

Figure S1. Large-scale screening of salt-alkali tolerant rice germplasms. (**A**) Growing of rice seedlings of different accessions in greenhouse on a large scale (594 rice accessions for each time) before SSAS. (**B**) Growing of 2376 rice seedlings of 99 accessions one by one in a big cultivating box containing the Yoshida's rice nutrient solution before SSAS. (**C**) Rice seedlings of different accessions treated with SSAS (140 mM $NaHCO_3$, pH 9.20) for 5 days. (**D**) Isolation and identification of the Salt-Alkali Tolerant Rice (SATR, indicated by the white arrow) and susceptible rice accession 93-11 (indicated by the blue arrow) by the large-scale screening system. SSAS, strong salt-alkali stress; d, day or days. Bar = 10 cm (**A**-**D**).

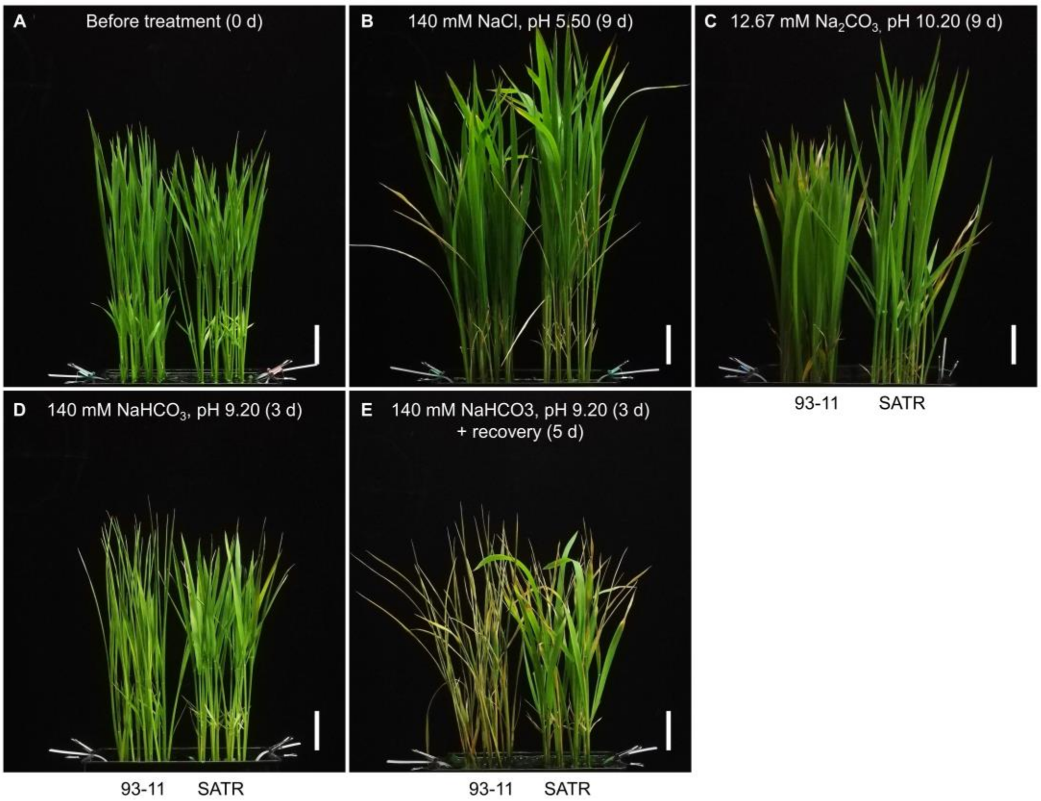

Figure S2. Evaluation of salt, alkali and salt-alkali tolerance between SATR and 93-11 seedlings. (**A**) SATR and 93-11 seedlings at 2-3 leaf stage before treatments (control). (**B**) SATR and 93-11 seedlings treated with single salt stress (140 mM NaCl, pH 5.50) for 9 days. (**C**) SATR and 93-11 seedlings treated with single alkali stress (approximately 12.67 mM Na_2CO_3, pH 10.20) for 9 days. (**D**) SATR and 93-11 seedlings treated with SSAS (140 mM $NaHCO_3$, pH 9.20) for 3 days. (**E**) SATR and 93-11 seedlings treated with SSAS for 3 days and subsequent 5 days of recovery. SATR, Salt-Alkali Tolerant Rice; SSAS, strong salt-alkali stress. d, day or days. Bar = 5 cm (**A**-**E**).

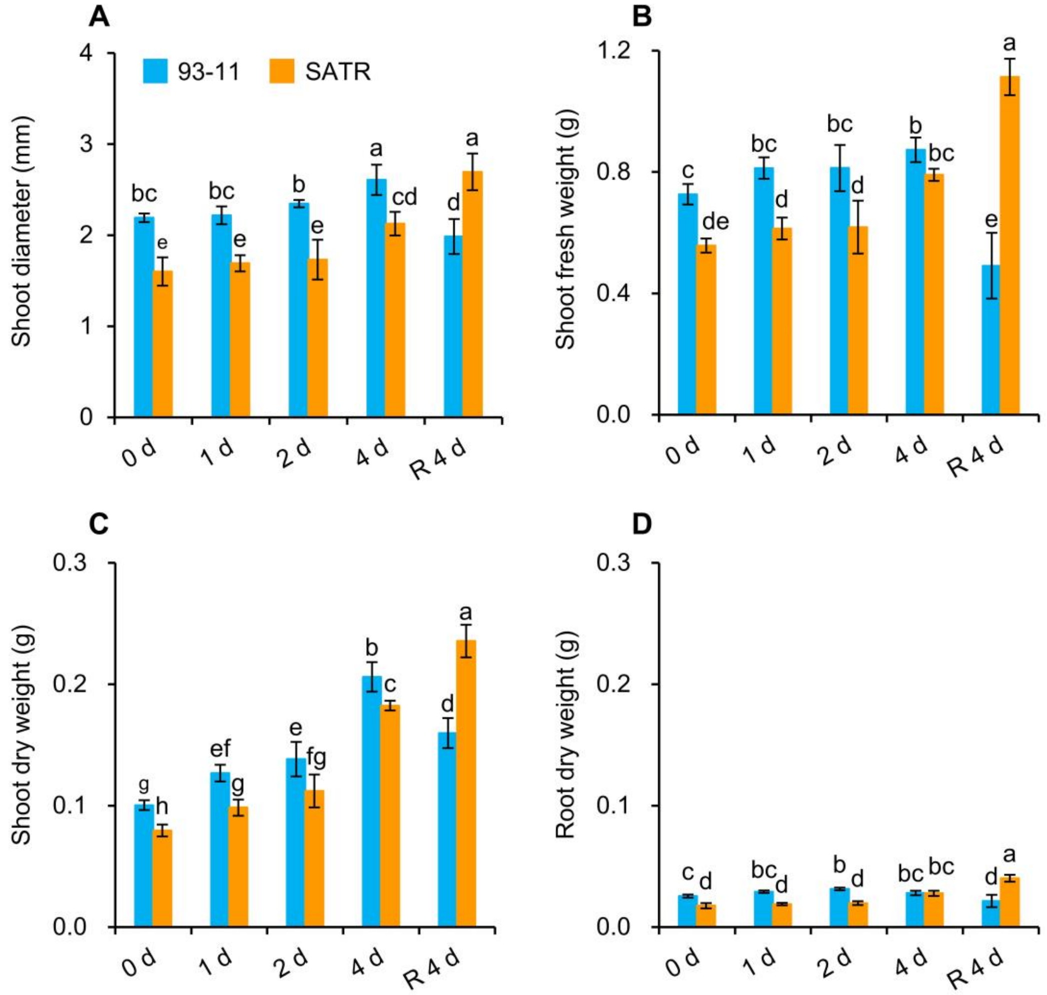

Figure S3. Phenotypic analysis in SATR and 93-11 seedlings under SSAS. (**A**-**D**) Comparisons of phenotypic indexes, including shoot diameter (**A**), shoot fresh weight (**B**), shoot dry weight (**C**), root dry weight (**D**) between SATR and 93-11 on the 0th, 1st, 2nd, 4th days after SSAS treatment and after 4 days of recovery, respectively. d, day or days; R 4 d, after 4 days of recovery. Data is given as mean ± SD, which was obtained from three independent biological replicates (**A**-**D**), and each replicate included 5 seedlings. The different letter on the bars in each bar chart indicates the significant difference at $P < 0.05$ obtained by one-way analysis of variance (ANOVA) and Duncan's new multiple range test.

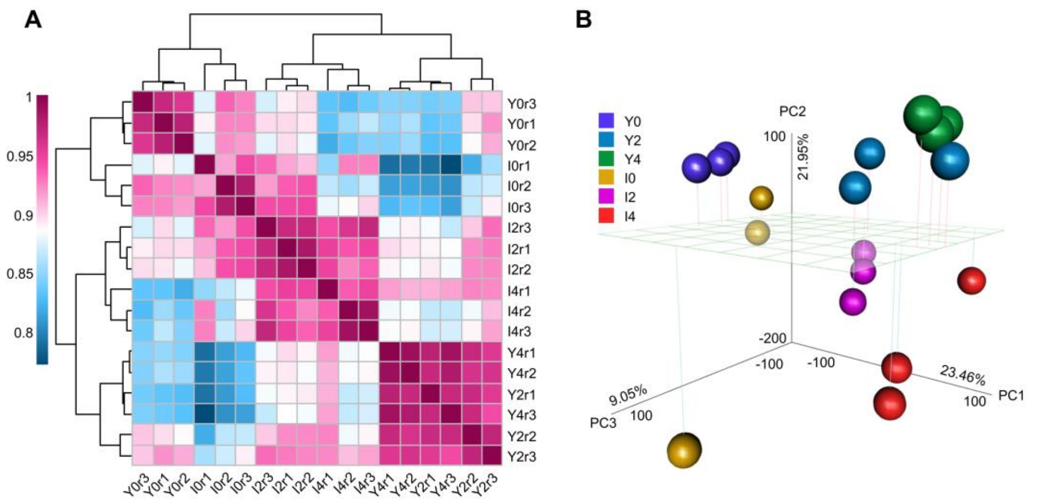

Figure S4. Pearson's correlation coefficient (PCC) and principal component analysis (PCA) of samples from SATR and 93-11 seedlings under SSAS. (**A**) Heat map of PCC analysis of sample-to-sample with respective FPKM values. (**B**) Three-dimensional diagram showing PCA results of samples. The purple (Y0), cyan (Y2) and green (Y4) balls indicate the samples of 93-11 seedlings on the 0th, 2nd and 4th days after SSAS treatment, respectively; the gold (I0), carmine (I2) and red (I4) balls indicate the samples of SATR seedlings on the 0th, 2nd and 4th days after SSAS treatment, respectively. Y0, Y2 and Y4 represent the samples of 93-11 on the 0th, 2nd and 4th days after SSAS treatment, respectively. I0, I2 and I4 represent the samples of SATR under SSAS for 0, 2 and 4 days, respectively. The r1, r2 and r3 represent three independent biological replicates, respectively. SATR, Salt-Alkali Tolerant Rice; SSAS, strong salt-alkali stress. Overall, the samples from SATR and 93-11 were clustered into two separated subgroups (SATR subgroup and 93-11 subgroup). Moreover, in the samples from both of the two subgroups, the three biological replicates were tightly correlated at each time point and showed relatively good similarity as a whole (**A**,**B**).

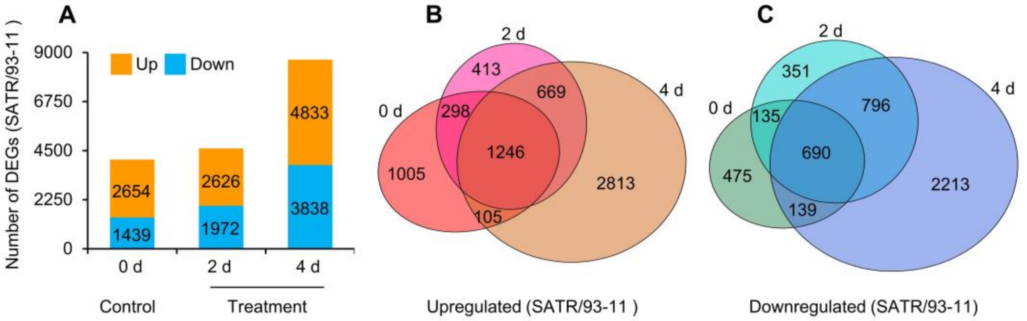

Figure S5. Statistical analysis of DEGs between SATR and 93-11 seedlings under SSAS. (**A**) Statistics of the number of significantly upregulated (yellow-brick bars) and downregulated (blue bars) DEGs between SATR and 93-11 on the 0th, 2nd and 4th days after SSAS treatment, respectively. (**B**,**C**) Venn diagrams show the distribution of unique, common and specific upregulated DEGs (warm colors) (**B**) and downregulated DEGs (cold colors) (**C**) between SATR and 93-11 on the 0th, 2nd and 4th days after SSAS treatment, respectively. The upregulated or downregulated DEGs were identified with the criteria of $|\log2 \text{ (fold change)}| > 1$ and corrected *P*-value (adjusted-*P*) < 0.05 (**A**-**C**). The number of DEGs corresponds to the size of each area in Venn diagrams (**B**,**C**). DEGs, differentially expressed genes; SATR, Salt-Alkali Tolerant Rice; SSAS, strong salt-alkali stress; d, day or days. A total of 5303 unique DEGs and 1246 common DEGs were identified from the comparison groups from upregulated DEGs (**B**). In contrast, 4799 unique DEGs and 690 common DEGs were identified from the comparison groups of downregulated DEGs (**C**). Besides, a total of 1005, 413 and 2813 specific DEGs were identified from the comparison groups of upregulated DEGs on the 0th, 2nd and 4th days after SSAS treatment, respectively (**B**). By comparison, 475, 351 and 2213 specific DEGs were identified from the comparison groups of downregulated DEGs on the 0th, 2nd and 4th days after SSAS treatment, respectively (**C**). There are more unique, common and specific DEGs in the

upregulated comparison groups than that in the corresponding downregulated comparison groups, which is consistent with the result that more upregulated DEGs were identified than downregulated DEGs under SSAS between SATR and 93-11 (**A**-**C**).

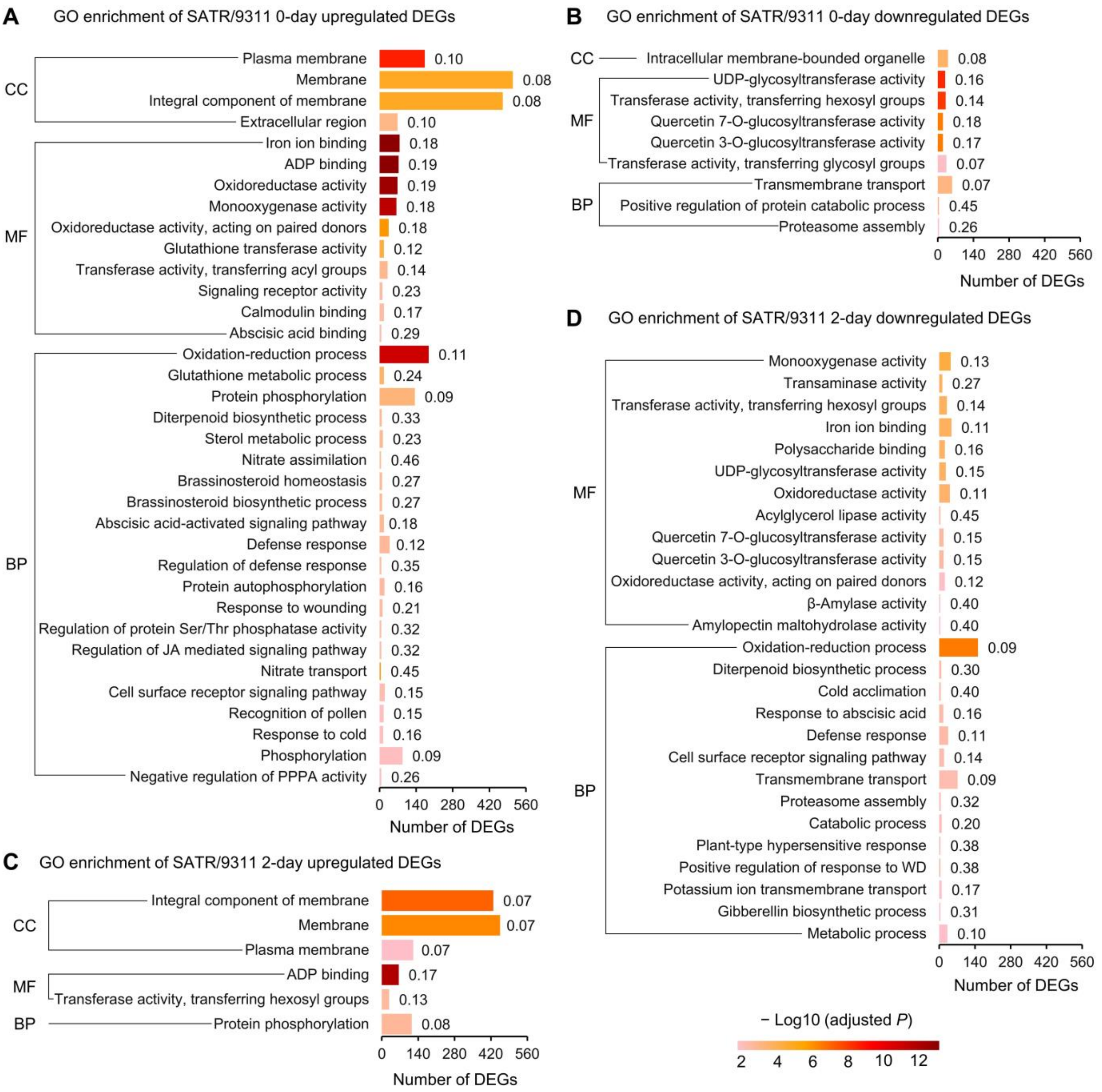

Figure S6. GO enrichment analysis of DEGs between SATR and 93-11 on the 0th and 2nd days after SSAS treatment. (**A** and **B**) Significantly enriched GO terms (adjusted-$P < 0.05$) from upregulated (**A**) and downregulated (**B**) DEGs between SATR and 93-11 under SSAS for 0 day in the three GO functional categories, consisting of cellular component (CC), molecular function (MF) and biological process (BP). With the cutoff of adjusted-$P < 0.05$, a total of 35 and 9 GO terms were significantly enriched from the upregulated (**A**) and downregulated (**B**) DEGs before SSAS (under SASS for 0 day), respectively. In the 35 significantly enriched GO terms from upregulated DEGs in normal growth conditions, plasma membrane and its integral components as well as extra-cellular region are the mainly enriched CC terms, oxidoreductase and monooxygenase activity are the dominantly MF terms, and defense response, plant hormone metabolic process and signaling pathway (abscisic acid and jasmonic acid signaling pathways) as well as oxidation-reduction process are the main BP terms (**A**). (**C**,**D**) Significantly enriched GO terms (adjusted-$P < 0.05$) from upregulated (**C**) and

downregulated (**D**) DEGs between SATR and 93-11 on the 2nd day after SSAS treatment in the GO functional categories. Besides, a total of 6 (**C**) and 27 (**D**) GO terms were significantly enriched from upregulated and downregulated DEGs on the 2nd day after SASS treatment, respectively. Three cell membrane-associated CC terms (integral component of membrane, membrane and plasma membrane) were significantly enriched from the upregulated DEGs on the 2nd day after SSAS treatment (**C**). The color of each bar and the color scale at the bottom correspond to the value of negative log10 (adjust-*P*), indicating the significance of GO terms enriched by the Fisher's exact test (the darker the color is, the higher significance of the corresponding GO term is). The number on the right side of each bar indicates the rich factor (Number of DEGs enriched in this GO term/number of total background genes in this GO term). The horizontal axis indicates the number of DEGs significantly enriched in the GO terms. GO, Gene Ontology; DEGs, differentially expressed genes; SATR, Salt-Alkali Tolerant Rice; SSAS, strong salt-alkali stress.

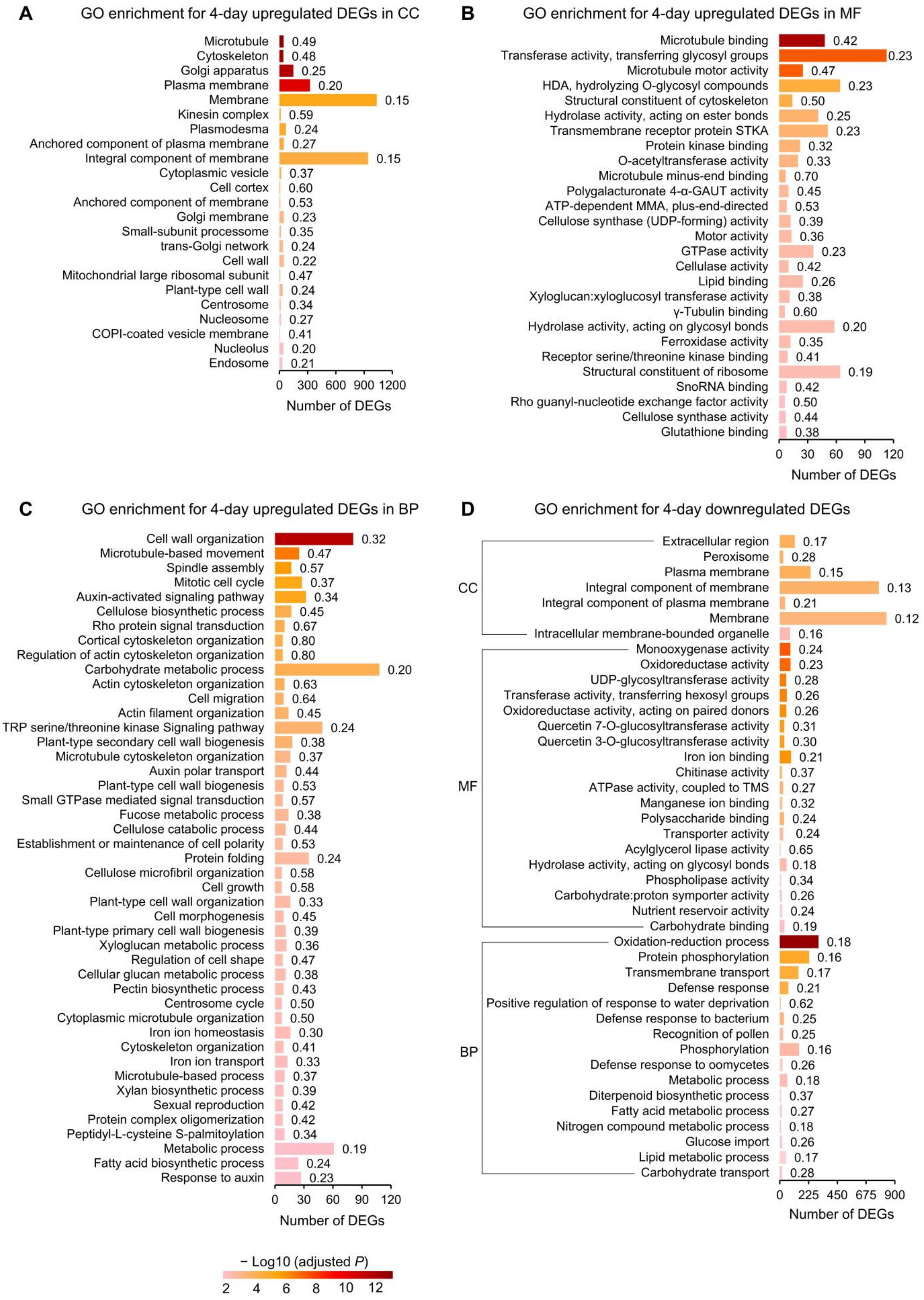
A
GO enrichment for 4-day upregulated DEGs in CC
Microtubule 0.49
Cytoskeleton 0.48
Golgi apparatus 0.25
Plasma membrane 0.20
Membrane 0.15
Kinesin complex 0.59
Plasmodesma 0.24
Anchored component of plasma membrane 0.27
Integral component of membrane 0.15
Cytoplasmic vesicle 0.37
Cell cortex 0.60
Anchored component of membrane 0.53
Golgi membrane 0.23
Small-subunit processome 0.35
trans-Golgi network 0.24
Cell wall 0.22
Mitochondrial large ribosomal subunit 0.47
Plant-type cell wall 0.24
Centrosome 0.34
Nucleosome 0.27
COPI-coated vesicle membrane 0.41
Nucleolus 0.20
Endosome 0.21
0 300 600 900 1200
Number of DEGs
B
GO enrichment for 4-day upregulated DEGs in MF
Microtubule binding 0.42
Transferase activity, transferring glycosyl groups 0.23
Microtubule motor activity 0.47
HDA, hydrolyzing O-glycosyl compounds 0.23
Structural constituent of cytoskeleton 0.50
Hydrolase activity, acting on ester bonds 0.25
Transmembrane receptor protein STKA 0.23
Protein kinase binding 0.32
O-acetyltransferase activity 0.33
Microtubule minus-end binding 0.70
Polygalacturonate 4-α-GAUT activity 0.45
ATP-dependent MMA, plus-end-directed 0.53
Cellulose synthase (UDP-forming) activity 0.39
Motor activity 0.36
GTPase activity 0.23
Cellulase activity 0.42
Lipid binding 0.26
Xyloglucan:xyloglucosyl transferase activity 0.38
γ-Tubulin binding 0.60
Hydrolase activity, acting on glycosyl bonds 0.20
Ferroxidase activity 0.35
Receptor serine/threonine kinase binding 0.41
Structural constituent of ribosome 0.19
SnoRNA binding 0.42
Rho guanyl-nucleotide exchange factor activity 0.50
Cellulose synthase activity 0.44
Glutathione binding 0.38
0 30 60 90 120
Number of DEGs
C
GO enrichment for 4-day upregulated DEGs in BP
Cell wall organization 0.32
Microtubule-based movement 0.47
Spindle assembly 0.57
Mitotic cell cycle 0.37
Auxin-activated signaling pathway 0.34
Cellulose biosynthetic process 0.45
Rho protein signal transduction 0.67
Cortical cytoskeleton organization 0.80
Regulation of actin cytoskeleton organization 0.80
Carbohydrate metabolic process 0.20
Actin cytoskeleton organization 0.63
Cell migration 0.64
Actin filament organization 0.45
TRP serine/threonine kinase Signaling pathway 0.24
Plant-type secondary cell wall biogenesis 0.38
Microtubule cytoskeleton organization 0.37
Auxin polar transport 0.44
Plant-type cell wall biogenesis 0.53
Small GTPase mediated signal transduction 0.57
Fucose metabolic process 0.38
Cellulose catabolic process 0.44
Establishment or maintenance of cell polarity 0.53
Protein folding 0.24
Cellulose microfibril organization 0.58
Cell growth 0.58
Plant-type cell wall organization 0.33
Cell morphogenesis 0.45
Plant-type primary cell wall biogenesis 0.39
Xyloglucan metabolic process 0.36
Regulation of cell shape 0.47
Cellular glucan metabolic process 0.38
Pectin biosynthetic process 0.43
Centrosome cycle 0.50
Cytoplasmic microtubule organization 0.50
Iron ion homeostasis 0.30
Cytoskeleton organization 0.41
Iron ion transport 0.33
Microtubule-based process 0.37
Xylan biosynthetic process 0.39
Sexual reproduction 0.42
Protein complex oligomerization 0.42
Peptidyl-L-cysteine S-palmitoylation 0.34
Metabolic process 0.19
Fatty acid biosynthetic process 0.24
Response to auxin 0.23
0 30 60 90 120
Number of DEGs
D
GO enrichment for 4-day downregulated DEGs
CC
Extracellular region 0.17
Peroxisome 0.28
Plasma membrane 0.15
Integral component of membrane 0.13
Integral component of plasma membrane 0.21
Membrane 0.12
Intracellular membrane-bounded organelle 0.16
MF
Monooxygenase activity 0.24
Oxidoreductase activity 0.23
UDP-glycosyltransferase activity 0.28
Transferase activity, transferring hexosyl groups 0.26
Oxidoreductase activity, acting on paired donors 0.26
Quercetin 7-O-glucosyltransferase activity 0.31
Quercetin 3-O-glucosyltransferase activity 0.30
Iron ion binding 0.21
Chitinase activity 0.37
ATPase activity, coupled to TMS 0.27
Manganese ion binding 0.32
Polysaccharide binding 0.24
Transporter activity 0.24
Acylglycerol lipase activity 0.65
Hydrolase activity, acting on glycosyl bonds 0.18
Phospholipase activity 0.34
Carbohydrate:proton symporter activity 0.26
Nutrient reservoir activity 0.24
Carbohydrate binding 0.19
BP
Oxidation-reduction process 0.18
Protein phosphorylation 0.16
Transmembrane transport 0.17
Defense response 0.21
Positive regulation of response to water deprivation 0.62
Defense response to bacterium 0.25
Recognition of pollen 0.25
Phosphorylation 0.16
Defense response to oomycetes 0.26
Metabolic process 0.18
Diterpenoid biosynthetic process 0.37
Fatty acid metabolic process 0.27
Nitrogen compound metabolic process 0.18
Glucose import 0.26
Lipid metabolic process 0.17
Carbohydrate transport 0.28
0 225 450 675 900
Number of DEGs
− Log10 (adjusted P)
2 4 6 8 10 12

Figure S7. GO enrichment analysis of DEGs between SATR and 93-11 on the 4th day after SSAS treatment. (**A**-**C**) Significantly enriched GO terms (adjusted-$P < 0.05$) from upregulated DEGs between SATR and 93-11 on the 4th day after SSAS treatment in the three GO functional categories, consisting of cellular component (CC) (**A**), molecular function (MF) (**B**) and biological process (BP) (**C**). (**D**) Significantly enriched GO terms (adjusted-$P < 0.05$) from downregulated DEGs between SATR and 93-11 on the 4th day after SSAS treatment in the GO functional categories of CC, MF and BP, respectively. The color of each bar and the color scale at the bottom corresponds to the value of negative log10 (adjust-*P*), indicating the significance of GO terms enriched by the Fisher's exact test (the darker the color is, the higher significance of the corresponding GO term is). The number on the right side of each bar indicates the rich factor (Number of DEGs enriched in this GO term/number of total background genes in this GO term. The horizontal axis indicates the number of DEGs significantly enriched in the GO terms. GO, Gene Ontology; DEGs, differentially expressed genes; SATR, Salt-Alkali Tolerant Rice; SSAS, strong salt-alkali stress. HDA, Hydrolase activity; MMA, microtubule motor activity; STKA, serine/threonine kinase activity. A total of 95 (**A**-**C**) and 42 (**D**) GO terms were significantly enriched from the upregulated and downregulated DEGs on the 4th day after SASS treatment, respectively. In addition, in the 95 significantly enriched GO terms from upregulated DEGs on the 4th day after SSAS treatment, cell wall, cell membrane and integral component, as well as cytoskeleton system including microtubule and kinesin complex are the highlighted CC terms (**A**). Besides, cytoskeleton system related molecular functions including structural constituent of cytoskeleton, microtubule and tubulin binding as well as microtubule motor activity, cellulose synthase and xyloglucosyl transferase activity are the dominant MF terms (**B**). Cell wall and cytoskeleton system related biological processes are the overwhelming BP terms, which accounts for 62.22% in the BP terms. Firstly, it contains BP terms of cell wall biogenesis and organization, cell polarity establishment or maintenance, cell growth and morphogenesis as well as migration, and cell shape regulation. Secondly, it also contains cell wall matrix polysaccharide biosynthesis and metabolism related BP terms including cellulose biosynthetic and catabolic processes, cellulose microfibril organization, xylan biosynthetic and xyloglucan metabolic processes, pectin biosynthetic processes, cellular glucan and fucose metabolic processes. Thirdly, the BP terms of cytoskeleton system organization and regulation, as well as microtubule-based process and movement are also included (**C**).

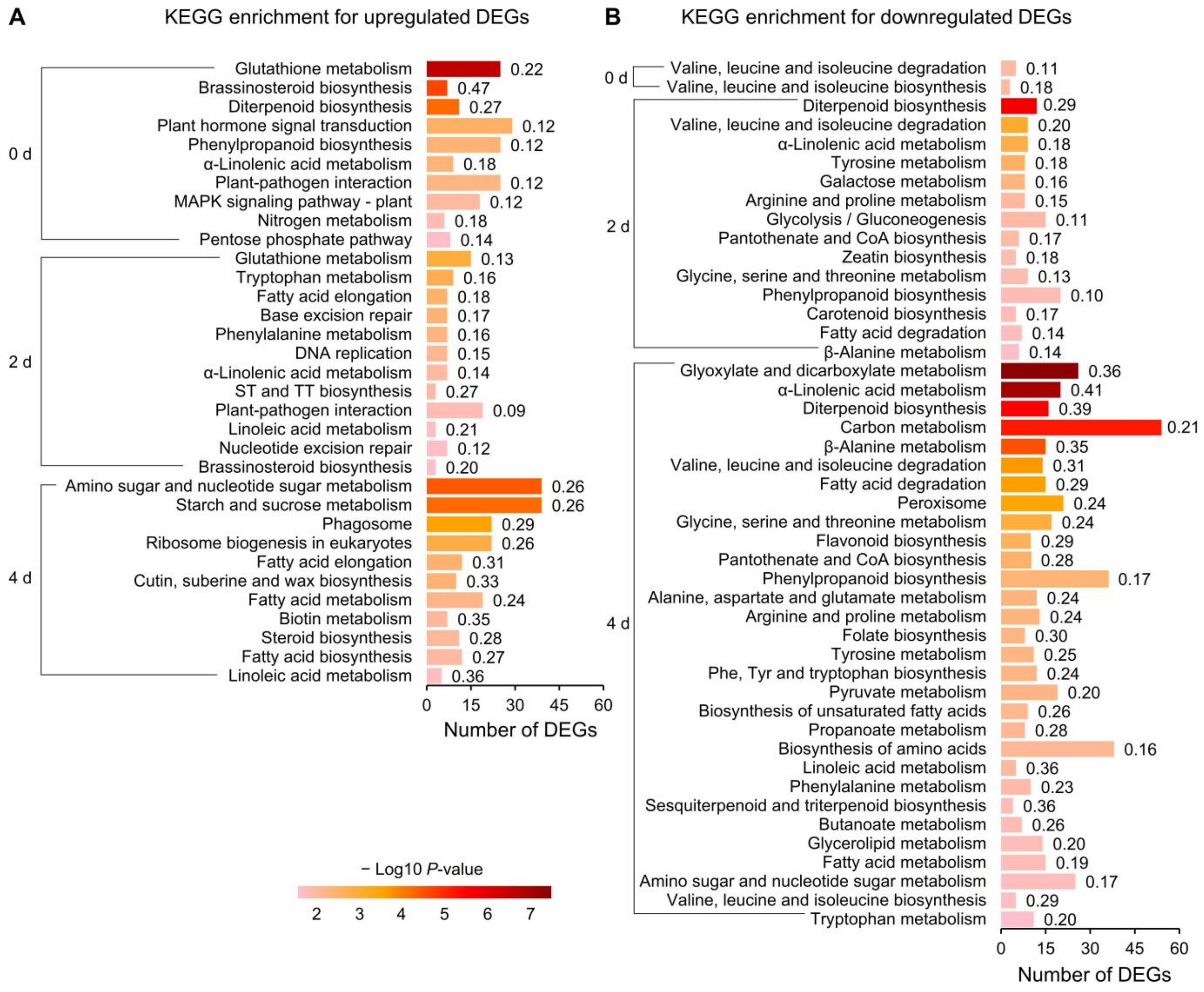

Figure S8. KEGG enrichment analysis of DEGs between SATR and 93-11 under SSAS. (**A**,**B**) Significantly enriched KEGG pathways (*P*-value < 0.05) from upregulated DEGs (**A**) and downregulated DEGs (**B**) between SATR and 93-11 on the 0th, 2nd and 4th days after SSAS treatment, respectively. The color of each bar and the color scale at the bottom corresponds to the value of negative log10 (*P*-value), indicating the significance of KEGG pathway enriched by the Fisher's exact test (the darker the color is, the higher significance of the corresponding KEGG pathway is). The number on the right side of each bar indicates the rich factor (number of DEGs enriched in this KEGG pathway/number of total background genes in this KEGG pathway). The horizontal axis indicates the number of DEGs significantly enriched in the KEGG pathways. KEGG, Kyoto Encyclopedia of Genes and Genomes; DEGs, differentially expressed genes; SATR, Salt-Alkali Tolerant Rice; SSAS, strong salt-alkali stress. ST, Sesquiterpenoid; TT, triterpenoid; d, day or days. With the cutoff of *P*-value < 0.05, a total of 33 (**A**) and 46 (**B**) KEGG pathways were significantly enriched from the upregulated (**A**) and downregulated (**B**) DEGs, respectively. It contains 10, 12 and 11 pathways on the 0th, 2nd and 4th days after SSAS treatment from the upregulated DEGs (**A**), as well as 2, 14 and 30 pathways on the 0th, 2nd and 4th days after SSAS treatment from the downregulated DEGs (**B**), respectively. There are the least (12) and the most (41) significantly enriched KEGG pathways on the 0th and 4th days after SSAS treatment, respectively (**A**,**B**). Glutathione metabolism, brassinosteroid biosynthesis, diterpenoid biosynthesis and plant hormone signal transduction are the top four significantly enriched KEGG pathways from upregulated DEGs in normal growth conditions (under SSAS for 0 day) (**A**).

Table S1. Summary of RNA sequencing outputs.

Sample[a]	Fastq file	Number of obtained Reads	Number of obtained bases	Number of Q20 bases (percentage of Q20 bases) [b]	Number of Q30 bases (percentage of Q30 bases) [c]	GC content	Number of fuzzy bases (percentage of fuzzy bases) [d]	Average read length (bp)
Y0r1	Y0r1.1.fq.gz	22756825	3413523750	3329851106 (97.55%)	3201065381 (93.78%)	60.78%	45357 (0.00%)	150
Y0r1	Y0r1.2.fq.gz	22756825	3413523750	3334364648 (97.68%)	3203737371 (93.85%)	60.70%	38340 (0.00%)	150
Y0r2	Y0r2.1.fq.gz	27727345	4159101750	4057439452 (97.56%)	3900130925 (93.77%)	61.27%	54748 (0.00%)	150
Y0r2	Y0r2.2.fq.gz	27727345	4159101750	4057880963 (97.57%)	3894063128 (93.63%)	61.22%	46082 (0.00%)	150
Y0r3	Y0r3.1.fq.gz	27535180	4130277000	4030121796 (97.58%)	3875297245 (93.83%)	60.63%	53857 (0.00%)	150
Y0r3	Y0r3.2.fq.gz	27535180	4130277000	4027020991 (97.50%)	3858216445 (93.41%)	60.60%	45719 (0.00%)	150
I0r1	I0r1.1.fq.gz	22869011	3430351650	3344097241 (97.49%)	3217818170 (93.80%)	60.37%	23697 (0.00%)	150
I0r1	I0r1.2.fq.gz	22869011	3430351650	3350295866 (97.67%)	3223314524 (93.96%)	60.24%	22251 (0.00%)	150
I0r2	I0r2.1.fq.gz	24929634	3739445100	3645718909 (97.49%)	3507957485 (93.81%)	61.15%	25745 (0.00%)	150
I0r2	I0r2.2.fq.gz	24929634	3739445100	3645550373 (97.49%)	3498027766 (93.54%)	61.06%	24567 (0.00%)	150
I0r3	I0r3.1.fq.gz	24103326	3615498900	3519460617 (97.34%)	3385232282 (93.63%)	61.80%	25225 (0.00%)	150
I0r3	I0r3.2.fq.gz	24103326	3615498900	3518530541 (97.32%)	3375324406 (93.36%)	61.70%	23361 (0.00%)	150
Y2r1	Y2r1.1.fq.gz	24245090	3636763500	3544237096 (97.46%)	3402217089 (93.55%)	59.70%	47794 (0.00%)	150
Y2r1	Y2r1.2.fq.gz	24245090	3636763500	3544760609 (97.47%)	3393716203 (93.32%)	59.62%	40387 (0.00%)	150
Y2r2	Y2r2.1.fq.gz	24418578	3662786700	3574608606 (97.59%)	3439875901 (93.91%)	60.45%	25536 (0.00%)	150
Y2r2	Y2r2.2.fq.gz	24418578	3662786700	3569912043 (97.46%)	3418549149 (93.33%)	60.39%	23563 (0.00%)	150
Y2r3	Y2r3.1.fq.gz	21156955	3173543250	3099413400 (97.66%)	2984745669 (94.05%)	60.44%	17041 (0.00%)	150
Y2r3	Y2r3.2.fq.gz	21156955	3173543250	3088656879 (97.33%)	2950521100 (92.97%)	60.38%	15446 (0.00%)	150
I2r1	I2r1.1.fq.gz	24414736	3662210400	3571670859 (97.53%)	3438559274 (93.89%)	60.43%	25023 (0.00%)	150
I2r1	I2r1.2.fq.gz	24414736	3662210400	3564988466 (97.35%)	3412883217 (93.19%)	60.36%	23167 (0.00%)	150
I2r2	I2r2.1.fq.gz	23449691	3517453650	3435381921 (97.67%)	3309805414 (94.10%)	60.30%	24392 (0.00%)	150
I2r2	I2r2.2.fq.gz	23449691	3517453650	3412983696 (97.03%)	3245878196 (92.28%)	60.26%	22648 (0.00%)	150
I2r3	I2r3.1.fq.gz	24946531	3741979650	3650869731 (97.57%)	3513413032 (93.89%)	60.47%	25849 (0.00%)	150
I2r3	I2r3.2.fq.gz	24946531	3741979650	3649507143 (97.53%)	3500966772 (93.56%)	60.39%	24583 (0.00%)	150
Y4r1	Y4r1.1.fq.gz	24871815	3730772250	3644251974 (97.68%)	3507529469 (94.02%)	60.10%	20142 (0.00%)	150
Y4r1	Y4r1.2.fq.gz	24871815	3730772250	3652255973 (97.90%)	3518045642 (94.30%)	60.05%	18268 (0.00%)	150
Y4r2	Y4r2.1.fq.gz	25501183	3825177450	3727891455 (97.46%)	3584933038 (93.72%)	60.47%	26408 (0.00%)	150
Y4r2	Y4r2.2.fq.gz	25501183	3825177450	3714222089 (97.10%)	3541544789 (92.59%)	60.41%	24370 (0.00%)	150
Y4r3	Y4r3.1.fq.gz	20127907	3019186050	2943136232 (97.48%)	2829427798 (93.71%)	59.80%	20871 (0.00%)	150
Y4r3	Y4r3.2.fq.gz	20127907	3019186050	2949139855 (97.68%)	2834930931 (93.90%)	59.66%	19580 (0.00%)	150
I4r1	I4r1.1.fq.gz	23781492	3567223800	3481767918 (97.60%)	3349637101 (93.90%)	58.12%	24250 (0.00%)	150
I4r1	I4r1.2.fq.gz	23781492	3567223800	3428610628 (96.11%)	3213414365 (90.08%)	58.05%	23099 (0.00%)	150
I4r2	I4r2.1.fq.gz	21867346	3280101900	3204224662 (97.69%)	3086296384 (94.09%)	58.97%	23044 (0.00%)	150
I4r2	I4r2.2.fq.gz	21867346	3280101900	3206057893 (97.74%)	3082674597 (93.98%)	58.96%	21168 (0.00%)	150
I4r3	I4r3.1.fq.gz	21046877	3157031550	3087212993 (97.79%)	2976625198 (94.29%)	59.66%	16955 (0.00%)	150
I4r3	I4r3.2.fq.gz	21046877	3157031550	3076332117 (97.44%)	2941715776 (93.18%)	59.63%	15351 (0.00%)	150

[a] Y0, Y2 and Y4 represent samples of the 93-11 seedlings subjected to strong salt-alkali stress (SSAS) on the 0th, 2nd and 4th days, respectively; I0, I2 and I4 represent samples of the salt-alkali tolerant rice (SATR) seedlings

subjected to SSAS on the 0th, 2nd and 4th days, respectively; r1, r2 and r3 represent three independent biological replicates, respectively.
[b] Number of bases with the base quality score ≥ Q20 (the percentage of the Q20 bases in total obtained bases).
[c] Number of bases with the base quality score ≥ Q30 (the percentage of the Q30 bases in total obtained bases).
[d] Number of fuzzy bases (the percentage of number of fuzzy bases in the total obtained bases).
More than 6 GB raw bases were obtained for each sample. And further quality control analysis showed that the percentages of Q20 and Q30 bases fell into the ranges of 96.11% - 97.90% and 90.08% - 94.30%, respectively.

Table S2. Statistics of the clean reads mapped to the rice reference genome

Sample [a]	Total records [b]	Unmapped reads [c]	Multiple mapped reads [d]	Unique mapped reads [e]	Unsplice mapped reads [f]	Splice mapped reads [g]	Proper pairs mapped reads [h]	Percentage of mapped reads	Percentage of unmapped reads
Y0r1	46556000	3800221	702519	41010910	28482202	12528708	38817676	91.65%	8.35%
Y0r2	56873396	4427919	867992	50158779	35355759	14803020	47446862	92.02%	7.98%
Y0r3	56396036	4488043	928053	49654264	34176819	15477445	47003156	91.85%	8.15%
I0r1	47016237	3264555	757688	41715779	29268583	12447196	39758498	92.86%	7.14%
I0r2	51131111	3337909	829418	45691941	32089332	13602609	43640062	93.31%	6.69%
I0r3	49456322	3527723	799755	43879174	31085657	12793517	41886676	92.68%	7.32%
Y2r1	49914215	4236444	801558	43452178	30235370	13216808	41067160	91.26%	8.74%
Y2r2	50146805	4098699	783171	43955286	30426659	13528627	41601238	91.61%	8.39%
Y2r3	43514210	3598886	692001	38023023	26342428	11680595	36007260	91.49%	8.51%
I2r1	49966438	3505255	720953	44603264	31230109	13373155	42658364	92.82%	7.18%
I2r2	48084380	3232530	760765	42906087	29649907	13256180	41045014	93.11%	6.89%
I2r3	51083602	3531591	750734	45610737	31863144	13747593	43580244	92.92%	7.08%
Y4r1	51221156	4347894	832457	44563279	31153338	13409941	42182450	91.26%	8.74%
Y4r2	52547123	5098398	844880	45059088	31887888	13171200	42503450	90.00%	10.00%
Y4r3	41504771	3779029	680893	35795892	24962253	10833639	33785510	90.61%	9.39%
I4r1	50116762	4036126	1182364	42344494	29060135	13284359	40333768	91.51%	8.49%
I4r2	45072759	3091610	750780	39892302	27613510	12278792	38326102	92.93%	7.07%
I4r3	43356967	3028323	728080	38337351	26653867	11683484	36699564	92.81%	7.19%

[a] Y0, Y2 and Y4 represent samples of the 93-11 seedlings subjected to strong salt-alkali stress (SSAS) on the 0th, 2nd and 4th days, respectively; I0, I2 and I4 represent samples of the salt-alkali stress rice (SATR) seedlings subjected to SSAS on the 0th, 2nd and 4th days, respectively; r1, r2 and r3 represent three independent biological replicates, respectively.
[b] Total number of aligned sequences.
[c] Number of reads unmapped to the reference genome.
[d] Number of reads mapped to the reference genome multiply.
[e] Number of reads mapped to the reference genome uniquely.
[f] Number of unspliced reads mapped to the reference genome.
[g] Number of spliced reads mapped to the reference genome.
[h] Number of reads mapped to the reference genome in pairs properly.

Table S3. Associated gene annotation in hemicellulose and pectin biosynthesis pathway in rice

Acronym of enzyme name	Enzyme name	Biochemical reaction/reactions catalyzed	Gene ID	Gene symbol	Description
HXK	hexokinase	alpha-D-Glucose (Glc) → alpha-D-Glucose 6-phosphate (Glc-6P)	*LOC_Os01g09460*		Similar to Hexokinase
			LOC_Os01g52450		Similar to Hexokinase
			LOC_Os01g53930	*OsHXK6*	Hexokinase 6, Glucose sensor, regulation of mitochondrial RNA metabolism
			LOC_Os01g71320		Similar to Hexokinase
			LOC_Os05g09500		Similar to Hexokinase
			LOC_Os05g44760	*OsHXK5*	Hexokinase 5
			LOC_Os07g26540		Similar to OSIGBa0102I15.5 protein
GPI	glucose-6-phosphate isomerase	β-D-Fructose 6-phosphate (Fru-6P) → alpha-D-Glucose 6-phosphate (Glc-6P)	*LOC_Os03g56460*		Glucose-6-phosphate isomerase, cytosolic A
			LOC_Os06g14510		Similar to glucose-6-phosphate isomerase
			LOC_Os08g37380		Phosphoglucose isomerase family protein
			LOC_Os09g29070		Similar to glucose-6-phosphate isomerase-like protein (Fragment)
PGM	phosphoglucomutase	alpha-D-Galactose 1-phosphate (Glc-1P) ↔ alpha-D-Glucose 6-phosphate (Glc-6P)	*LOC_Os03g50480*	*OscPGM*	Cytosolic phosphoglucomutase
			LOC_Os10g11140	*OspPGM*	Plastidic phosphoglucomutase, starch synthesis in rice pollen
AGPL	UDP-glucose-hexose-1-phosphate uridylyl transferase	ADP-alpha-D-glucose (ADP-Glc) ↔ alpha-D-Galactose 1-phosphate (Glc-1P)	*LOC_Os01g44220*	*OsAGPL2*	ADP-glucose pyrophosphorylase large subunit
			LOC_Os03g52460	*OsAGPL3*	Chloroplastic/amyloplastic ADP-glucose pyrophosphorylase large subunit
			LOC_Os05g50380	*OsAGPL1*	Chloroplastic/amyloplastic ADP-glucose pyrophosphorylase large subunit
			LOC_Os08g25734	*OsAGPS2b*	Cytosolic ADP-glucose pyrophosphorylase small subunit 2b
			LOC_Os09g12660	*OsAGPS1*	Chloroplastic/amyloplastic ADP-glucose pyrophosphorylase small subunit 1
UGP	UTP-glucose-1-phosphate uridylyl transferase	alpha-D-Galactose 1-phosphate (Glc-1P)	*LOC_Os01g15910*		Similar to UDP-glucose pyrophosphorylase

		↔ UDP-glucose (UDP-Glc)	*LOC_Os02g02560*	*OsUgp2*	UDPase
			LOC_Os09g38030	*OsUgp1*	UDPase, pollen mother cell meiosis, pollen development
USP	UDP-sugar pyro phosphorylase	alpha-D-Galactose 1-phosphate (Glc-1P) ↔ UDP-glucose (UDP-Glc); UDP-alpha-D-galactose (UDP-Gal) ↔ alpha-D-Galactose 1-phosphate (Gal-1P)	*LOC_Os06g48760*		UTP-glucose-1-phosphate uridylyltransferase family protein
UGE	UDP-glucose 4-epimerase	UDP-glucose (UDP-Glc) ↔ UDP-alpha-D-galactose (UDP-Gal)	*LOC_Os08g28730*	*OsUGE2*	UDP-glucose epimerase, modulation of cellulose biosynthesis and wall assembly
			LOC_Os09g15420		Similar to UDP-galactose 4-epimerase-like protein
			LOC_Os09g35800		Similar to UDP-glucose 4-epimerase
UGHPU	UDP-glucose-hexose-1-phosphate uridylyl transferase	UDP-alpha-D-galactose (UDP-Gal) ↔ alpha-D-Galactose 1-phosphate (Gal-1P)	*LOC_Os07g07550*		Similar to galactose-1-phosphate uridyl transferase-like protein
UG46D	UDP-glucose 4,6-dehydratase	UDP-glucose (UDP-Glc) → UDP-4-dehydro-6-deoxy-D-glucose (UDP-DDG)	*LOC_Os03g17000*		NAD(P)-binding domain containing protein
ER	3,5-epimerase/4-reductase	UDP-4-dehydro-6-deoxy-D-glucose (UDP-DDG) → UDP-4-keto-rhamnose → UDP-L-rhamnose (UDP-Rha)	*LOC_Os02g45540*		NAD(P)-binding domain containing protein
UG6D	UDP-glucose 6-dehydrogenase	UDP-glucose (UDP-Glc) → UDP-D-glucuronate (UDP-GlcA)	*LOC_Os03g40720*		Similar to UDP-glucose dehydrogenase
			LOC_Os03g55070		Similar to UDP-glucose 6-dehydrogenase
			LOC_Os12g25690		Similar to UDP-glucose 6-dehydrogenase
			LOC_Os12g25700		Similar to UDP-glucose 6-dehydrogenase
UGUE	UDP-glucuronate 4-epimerase	UDP-D-glucuronate (UDP-GlcA) ↔ UDP-D-galacturonate (UDP-GalA)	*LOC_Os02g54890*		Similar to nucleotide sugar epimerase-like protein
			LOC_Os03g14540		Similar to nucleotide sugar epimerase-like protein

			LOC_Os08g41440		NAD(P)-binding domain containing protein
			LOC_Os09g32670		Similar to nucleotide sugar epimerase-like protein
GAUT	alpha-1,4-galacturonosyltransferase	UDP-D-galacturonate (UDP-GalA) → Poly(1,4-alpha-D-galacturonide) (Pectin)	*LOC_Os02g29530*		Polygalacturonate 4-alpha-galacturonosyltransferase/transferase
			LOC_Os02g51130		Glycosyl transferase, family 8 protein
			LOC_Os03g21250		Glycosyl transferase, family 8 protein
			LOC_Os03g30000		Glycosyl transferase, family 8 protein
			LOC_Os04g54360		Glycosyl transferase, family 8 protein
			LOC_Os06g12280		Hypothetical conserved gene
			LOC_Os06g49810		Similar to glycosyltransferase QUASIMODO1
			LOC_Os06g51160		Similar to glycosyltransferase, family GT8
			LOC_Os07g48370		Glycosyl transferase, family 8 protein
			LOC_Os08g23780	*OsGAUT4*	Galacturonosyltransferase, Pectin biosynthesis
			LOC_Os09g30280		Similar to glycosyltransferase QUASIMODO1
			LOC_Os09g36180		Glycosyl transferase, family 8 protein
			LOC_Os09g36190		Similar to glycosyltransferase QUASIMODO1
			LOC_Os11g03160		Glycosyl transferase, family 8 protein
			LOC_Os11g37980		Glycosyl transferase, family 8 protein
UXS	UDP-glucuronate decarboxylase	UDP-D-glucuronate (UDP-GlcA) → UDP-alpha-D-xylose (UDP-D-Xyl)	*LOC_Os01g21320*		UDP-glucuronic acid decarboxylase
			LOC_Os01g62020		Similar to UDP-glucuronic acid decarboxylase 1
			LOC_Os03g16980		UDP-glucuronic acid decarboxylase
			LOC_Os03g17230		Similar to UDP-D-glucuronate decarboxylase
			LOC_Os05g29990		UDP-glucuronic acid decarboxylase

			LOC_Os07g47700		Hypothetical conserved gene
AXS	UDP-apiose/xylose synthase	UDP-D-glucuronate (UDP-GlcA) → UDP-D-apiose (UDP-D-Api); UDP-D-glucuronate (UDP-GlcA) → UDP-alpha-D-xylose (UDP-D-Xyl)	*LOC_Os01g73790*		NAD(P)-binding domain containing protein
UXE	UDP-arabinose 4-epimerase	UDP-alpha-D-xylose (UDP-D-Xyl) ↔ UDP-β-L-arabinopyranose (UDP-L-Ara*p*)	*LOC_Os04g52730*		Similar to cDNA clone: J013046D23, full insert sequence
			LOC_Os07g04690		NAD(P)-binding domain containing protein
			LOC_Os08g03570		NAD(P)-binding domain containing protein
UAM	UDP-arabinopyranose mutase	UDP-β-L-arabinopyranose (UDP-L-Ara*p*) ↔ UDP-L-arabinofuranose (UDP-L-Ara*f*)	*LOC_Os03g40270*		Reversibly glycosylated polypeptide
			LOC_Os04g56520		Similar to amylogenin; reversibly glycosylatable polypeptide
			LOC_Os07g41360	*OsUAM3*	UDP-arabinopyranose mutase, pollen wall morphogenesis
OsCSLD4	1,4-β-D-xylan synthase	UDP-alpha-D-xylose (UDP-D-Xyl) → (1,4-β-D-xylan)n (1,4-β-D-Xylan)	*LOC_Os12g36890*	*OsCSLD4*	Cellulose synthase-like protein, cell wall biosynthesis
XBX	xylan-1,4-β-xylosidase	(1,4-β-D-xylan)n (1,4-β-D-Xylan) → D-Xylose (D-Xyl)	*LOC_Os01g19220*		Glycoside hydrolase, family 3, N-terminal domain containing protein
			LOC_Os04g54810		Similar to alpha-L-arabinofuranosidase/beta-D-xylosidase isoenzyme ARA-I

Table S4. Associated gene annotation in cutin, suberine and wax biosynthetic pathway in rice

Acronym of enzyme name	Enzyme name	Biochemical reaction catalyzed	Gene ID	Description
MAT	[acyl-carrier-protein] S-malonyl-transferase	[Acyl-carrier protein] (ACP) +Malonyl coenzyme A (Malonyl-CoA) → Malonyl-[acyl-carrier protein] (Malonyl-ACP)	*LOC_Os03g18590*	Acyl transferase domain containing protein
OASIII	3-oxoacyl-[acyl-carrier-protein] synthase III	Malonyl-[acyl-carrier protein] (Malonyl-ACP) + Acyl coenzyme A (Acyl-CoA) → Acetoacetyl-[acyl-carrier protein] (3-Oxoacyl-ACP)	*LOC_Os04g55060*	Similar to 3-ketoacyl carrier protein synthase III
OASII	3-oxoacyl-[acyl-carrier-protein] synthase II	Malonyl-[acyl-carrier protein] (Malonyl-ACP) + Acyl-[acyl-carrier protein] (LC acyl-ACP) → Acetoacetyl-[acyl-carrier protein] (3-Oxoacyl-ACP)	*LOC_Os02g10320*	Similar to 3-oxoacyl-[acyl-carrier-protein] synthase
			LOC_Os04g36800	Similar to osigba0140o078 protein
			LOC_Os06g09630	Beta-ketoacyl-[acyl carrier protein] synthase I, root development
			LOC_Os07g42420	Similar tobeta-ketoacyl-ACP synthase
OAR	3-oxoacyl-[acyl-carrier protein] reductase	Acetoacetyl-[acyl-carrier protein] (3-Oxoacyl-ACP) ↔ (R)-3-Hydroxyacyl-[acyl-carrier protein] ((R)-3-Hydroxy-acyl-ACP)	*LOC_Os02g30060*	Similar to 3-oxoacyl-[acyl-carrier-protein] reductase, chloroplast precursor
			LOC_Os03g53690	Similar to chort-chain type dehydrogenase/reductase
			LOC_Os04g30760	3-Oxoacyl-reductase, cuticular wax biosynthesis and chloroplast development
			LOC_Os06g08600	Similar to estradiol 17-beta-dehydrogenase 8
			LOC_Os07g07440	Similar to Brn1-like protein
			LOC_Os07g37420	Short-chain dehydrogenase/reductase SDR domain containing protein
			LOC_Os10g31780	Similar to Brn1-like protein
HAD	3-hydroxyacyl-[acyl-carrier-protein] dehydratase	(R)-3-Hydroxyacyl-[acyl-carrier protein] ((R)-3-Hydroxy-acyl-ACP) ↔ *trans*-2-Enoyl-[acyl-carrier protein] (*t*-2-Enoyl-ACP)	*LOC_Os05g36000*	Similar to (3R)-hydroxymyristoyl-[acyl carrier protein] dehydratase
			LOC_Os08g12840	Non-protein coding transcript
EAR	enoyl-[acyl-carrier protein] reductase I	*trans*-2-Enoyl-[acyl-carrier protein] (t-2-Enoyl-ACP) ↔ Acyl-[acyl-carrier protein] (LC acyl-ACP)	*LOC_Os08g23810*	Similar to Enoyl-ACP reductase (Fragment)
			LOC_Os09g10600	Similar to Enoyl-[acyl-carrier-protein] reductase [NADH]
FATB	fatty acyl-ACP thioesterase B		*LOC_Os02g43090*	Similar to Acyl-ACP thioesterase (Fragment)

		Acyl-[acyl-carrier protein] (LCacyl-ACP) ↔ Long-chain fatty acid (LCFA)	*LOC_Os06g05130*	Similar to Acyl-ACP thioesterase (Fragment)
			LOC_Os06g39520	Similar to Acyl-ACP thioesterase (Fragment)
			LOC_Os11g43820	Similar to Acyl-ACP thioesterase (Fragment)
LACS	long-chain acyl-CoA synthetase	Long-chain fatty acid (LCFA) → Long-chain acyl-CoA (LC acyl-CoA) (n ≥ 16)	*LOC_Os01g46750*	Similar to ACS-like protein
			LOC_Os01g48910	Similar to Acyl-CoA synthetase
			LOC_Os03g62850	Similar to Acyl-CoA synthetase-like protein
			LOC_Os05g04170	AMP-dependent synthetase and ligase domain containing protein
			LOC_Os05g25310	AMP-dependent synthetase and ligase domain containing protein
			LOC_Os06g06350	Similar to Acyl-CoA synthetase
			LOC_Os11g04980	Similar to long chain acyl-CoA synthetase 6
			LOC_Os11g06880	Similar to long-chain-fatty-acid-CoA ligase 4
			LOC_Os11g35400	Similar to acyl CoA synthetase
			LOC_Os12g04990	Similar to long chain acyl-CoA synthetase 6
			LOC_Os12g07110	Long-chain acyl-CoA synthetase, starch degradation, plant growth
MEAR	mitochondrial enoyl-[acyl-carrier protein] reductase/*trans*-2-enoyl-CoA reductase	Acyl coenzyme A (Acyl-CoA) (n+2) ↔ (3S)-3-Hydroxyacyl-CoA (3-HOA-CoA)	*LOC_Os12g01160*	Alcohol dehydrogenase superfamily, zinc-containing protein
KCS	3-ketoacyl-CoA synthase	Long-chain acyl-CoA (LC acyl-CoA) (n ≥ 16) + Malonyl coenzyme A (Malonyl-CoA) → Very-long-chain 3-oxoacyl-CoA (VLC 3-OA-CoA)	*LOC_Os01g34560*	Similar to very-long-chain fatty acid condensing enzyme CUT1
			LOC_Os02g11070	Similar to fatty acid elongase 1
			LOC_Os02g49920	Similar to 3-ketoacyl-CoA synthase
			LOC_Os02g56860	Thiolase-like, subgroup domain containing protein
			LOC_Os03g06700	Similar to fatty acid elongase
			LOC_Os03g06705	Similar to beta-ketoacyl-CoA-synthase

			LOC_Os03g08360	Fatty acid elongase (beta-ketoacyl-CoA synthase), shoot development
			LOC_Os03g12030	Beta-ketoacyl-CoA synthase, homolog of Arabidopsis CER6
			LOC_Os03g14170	Thiolase-like, subgroup domain containing protein
			LOC_Os03g26530	Thiolase-like, subgroup domain containing protein
			LOC_Os04g02640	Similar to 3-ketoacyl-CoA synthase
			LOC_Os05g49290	Similar to FAE1
			LOC_Os05g49900	Similar to 3-ketoacyl-CoA synthase
			LOC_Os06g14810	Similar to 3-ketoacyl-CoA synthase
			LOC_Os06g15170	Similar to very-long-chain fatty acid condensing enzyme CUT1
			LOC_Os06g39750	Similar to fatty acid elongase 1-like protein
			LOC_Os09g19650	Thiolase-like, subgroup domain containing protein
			LOC_Os10g28060	Fatty acid elongase (beta-ketoacyl-CoA synthase), shoot development
			LOC_Os10g33370	Thiolase-like, subgroup domain containing protein
			LOC_Os11g37900	Similar to 3-ketoacyl-CoA synthase
KCR	17 beta-estradiol 17-dehydrogenase/very-long-chain 3-oxoacyl-CoA reductase	Very-long-chain 3-oxoacyl-CoA (VLC 3-OA-CoA) → Very-long-chain (3R)-3-hydroxyacyl-CoA (VLC 3-HOA-CoA)	*LOC_Os04g02620*	Glucose/ribitol dehydrogenase family protein
			LOC_Os04g40730	Beta-ketoacyl-CoA reductase, cuticular wax biosynthesis, fatty acid elongation
			LOC_Os06g19530	Glucose/ribitol dehydrogenase family protein
PAS	very-long-chain (3R)-3-hydroxyacyl-CoA dehydratase	Very-long-chain (3R)-3-hydroxyacyl-CoA (VLC 3-HOA-CoA) → Very-long-chain *trans*-2,3-dehydroacyl-CoA (VLC 2,3-DHOA-CoA)	*LOC_Os01g05694*	Protein-tyrosine phosphatase-like, PTPLA domain containing protein
			LOC_Os01g05710	Similar to 3-hydroxyacyl-CoA dehydratase PASTICCINO 2A
			LOC_Os01g05744	Protein-tyrosine phosphatase-like, PTPLA domain containing protein
			LOC_Os04g20280	Protein-tyrosine phosphatase-like, PTPLA domain containing protein
			LOC_Os05g38590	Protein-tyrosine phosphatase-like, PTPLA domain containing protein

CER10	very-long-chain enoyl-CoA reductase	Very-long-chain *trans*-2,3-dehydroacyl-CoA (VLC 2,3-DHOA-CoA) → Very-long-chain acyl-CoA (VL Cacyl-CoA)	*LOC_Os01g05670*	Similar to Synaptic glycoprotein SC2
ACAT	acyl-coenzyme A thioesterase 1/2/4	Very-long-chain acyl-CoA (VL Cacyl-CoA) → Long-chain fatty acid (LCFA)	*LOC_Os04g47120*	Rmlc-like jelly roll fold domain containing protein
CYP86	long-chain fatty acid omega-monooxygenase	Long-chain fatty acid (LCFA) → ω-Hydroxy fatty acid (16-HPA)	*LOC_Os01g63540*	Similar to Cytochrome P450 86A1
			LOC_Os02g44654	Similar to Cytochrome P450
			LOC_Os04g47250	Cytochrome P450 family protein
			LOC_Os10g34480	Cytochrome P450-like protein (CYP86B1)
CYP704B1	long-chain fatty acid omega-monooxygenase	Long-chain fatty acid (LCFA) → ω-Hydroxy fatty acid (16-HPA)	*LOC_Os03g07250*	Cytochrome P450 protein, anther cutin biosynthesis and pollen exine formation
FOHD	fatty acid omega-hydroxy dehydrogenase	ω-Hydroxy fatty acid (16-HPA) → ω-Oxo fatty acid (ω-OFA)	*LOC_Os08g31030*	Similar to HOTHEAD protein precursor (ADHESION OF CALYX EDGES protein)
			LOC_Os09g19930	Putative omega-alcohol dehydrogenase, biosynthesis of long-chain fatty acids
POA	peroxygenase	ω-Hydroxy fatty acid (16-HPA) → ω-Hydroxy epoxy-fatty acid (ω-HOEFA)	*LOC_Os03g12230*	Similar to caleosin
			LOC_Os04g43070	Conserved hypothetical protein
			LOC_Os06g14324	Similar to EF-hand calcium bining protein
			LOC_Os06g14350	Caleosin related family protein
			LOC_Os06g14370	Caleosin related family protein
CYP77A	cytochrome P450 family 77 subfamily A	ω-Hydroxy fatty acid (16-HPA) →Polyhydroxy fatty acid (PHOFA)	*LOC_Os04g33320*	Similar to Cytochrom-P450-like protein
			LOC_Os06g46680	Cytochrome P450 family protein
DPW	alcohol-forming fatty acyl-CoA reductase	Long-chain acyl-CoA (n ≥ 18) → Long-chain primary alcohol (LC Primary alcohol)	*LOC_Os03g07140*	Similar to Male sterility protein 2
			LOC_Os04g28520	Similar to osigba0092g141 protein
			LOC_Os04g28620	Similar to acyl CoA reductase-like protein
			LOC_Os08g20200	Similar to male sterility protein 2

			LOC_Os08g44360	Similar to fatty acyl CoA reductase
			LOC_Os09g39410	Similar to fatty acyl CoA reductase
CER1	aldehyde decarbonylase	Long-chain aldehyde (LC aldehyde) → Long-chain alkane (LC Alkane)	*LOC_Os02g40784*	Homologous protein of CER1, very-long-chain alkane biosynthesis
			LOC_Os02g56920	Similar to CER1-like gene protein (Fragment)
			LOC_Os10g33250	Protein with high similarity to proteins involved in wax production, cuticular wax biosynthesis

Table S5. Gene annotation of highlighted potential candidate genes controlling salt-alkali tolerance in SATR

category	Gene ID	Gene symbol (encoding protein)	chromosome number	Start	End	Description	Reference
Cell wall biogenesis associated genes	*LOC_Os03g52460*	*OsAGPL1* (AGPL)	3	30099369	30104596	Glucose-1-phosphate adenylyltransferase large subunit, putative, expressed	
	LOC_Os03g52630	*OsGH9A3*	3	30181636	30185153	Endoglucanase, putative, expressed	[1]
	LOC_Os03g55070	*LOC_Os03g55070* (UG6D)	3	31315805	31318395	UDP-glucose 6-dehydrogenase, putative, expressed	
	LOC_Os03g56460	*LOC_Os03g56460* (GPI)	3	32175133	32182101	Glucose-6-phosphate isomerase, putative, expressed	
	LOC_Os03g56810	*pTUB22* (β-Tubulin)	3	32371758	32374910	Tubulin/FtsZ domain containing protein, putative, expressed	
	LOC_Os03g56950	*OsPIL13*	3	32432456	32435845	Phytochrome-interacting factor 4, putative, expressed	[2]
	LOC_Os08g25734	*OsAGPS2a* (AGPL)	8	15666201	15672594	Glucose-1-phosphate adenylyltransferase large subunit, chloroplast precursor, putative, expressed	
	LOC_Os08g28730	*OsUGE2* (UGE)	8	17569906	17576414	NAD dependent epimerase/dehydratase family protein, putative, expressed	
	LOC_Os09g32080	*OsCTL1*	9	19148039	19150047	CHIT13 - Chitinase family protein precursor, expressed	[3]
	LOC_Os10g28060	*ONI2* (KCS)	10	14560601	14565737	3-Ketoacyl-CoA synthase, putative, expressed	

	LOC_Os10g31780	*LOC_Os10g31780* (OAR)	10	16663932	16664717	Oxidoreductase, short chain dehydrogenase/reductase family domain containing protein, expressed	
	LOC_Os10g31910	*DEL1*	10	16755564	16760878	Pectate lyase precursor, putative, expressed	[4]
	LOC_Os10g32980	*OsCesA7*	10	17261952	17266613	CESA7 - cellulose synthase, expressed	[5]
	LOC_Os10g33370	*LOC_Os10g33370* (KCS)	10	17564167	17571574	3-Ketoacyl-CoA synthase precursor, putative, expressed	
	LOC_Os10g34480	*LOC_Os10g34480* (CYP86)	10	18389977	18394398	Cytochrome P450, putative, expressed	
	LOC_Os10g36848	*OsCAld5H1*	10	19728254	19735041	Cytochrome P450, putative, expressed	[6]
	LOC_Os10g40710	*OsEXPB2*	10	21835930	21837651	Expansin precursor, putative, expressed	[7]
	LOC_Os10g40720	*OsEXPB3*	10	21847804	21849726	Expansin precursor, putative, expressed	[8]
	LOC_Os10g40960	*RL14*	10	21995563	21997584	Oxidoreductase, 2OG-Fe oxygenase family protein, putative, expressed	[9]
	LOC_Os10g42750	*OsCSLD1*	10	23062454	23066292	CSLD1 - cellulose synthase-like family D, expressed	[10]
Genes reported to directly control or be related to salt stress, osmotic stress or drought stress	*LOC_Os03g52650*	*OsKNOLLE*	3	30192884	30194132	Syntaxin-related protein, putative, expressed	[11]
	LOC_Os03g52690	*OsCBSX4*	3	30206350	30210436	CBS domain containing membrane protein, putative, expressed	[12]
	LOC_Os03g53020	*OsbHLH148*	3	30406830	30409447	Helix-loop-helix DNA-binding domain containing protein, expressed	[13]
	LOC_Os03g53500	*OsSUV3*	3	30679685	30689230	Helicase conserved C-terminal domain containing protein, expressed	[14]
	LOC_Os03g55290	*OsGASR1*	3	31464840	31465625	GASR3 - Gibberellin-regulated GASA/GAST/Snakin family protein precursor, expressed	[8]
	LOC_Os03g55600	*OsSAPK8*	3	31652694	31658253	CAMK_CAMK_like.22 - CAMK includes calcium/calmodulin depedent protein kinases, expressed	[15]
	LOC_Os03g56790	*OsADF2*	3	32360572	32363839	Actin-depolymerizing factor, putative, expressed	[15]
	LOC_Os03g58390	*OsSIRP2*	3	33267192	33272302	Zinc finger, C3HC4 type domain containing protein, expressed	[16]
	LOC_Os08g25570	*HDA705*	8	15554139	15559152	Histone deacetylase, putative, expressed	[17]

LOC_Os08g28710	*OsRLCK253*	8	17558062	17560192	Receptor protein kinase CRINKLY4 precursor, putative, expressed	[18]
LOC_Os10g28000	*OsGR3*	10	14505709	14508788	Glutathione reductase, putative, expressed	[19]
LOC_Os10g29560	*OsPQT3*	10	15360564	15369094	Transposon protein, putative, CACTA, En/Spm sub-class, expressed	[20]
LOC_Os10g33240	*OsDUF810.7*	10	17437474	17441023	Expressed protein	[21]
LOC_Os10g37660	*OsTRE1*	10	20154546	20159181	Trehalase precursor, putative, expressed	[22]
LOC_Os10g38489	*OsGSTU4*	10	20553664	20560790	Glutathione S-transferase GSTU6, putative, expressed	[23]

The AGPL, UGE, UG6D and GPI represent UDP-glucose-hexose-1-phosphate uridylyl transferase, UDP-glucose 4-epimerase, UDP-glucose 6-dehydrogenase and glucose-6-phosphate isomerase respectively, which all participate in the hemicellulose and pectin biosynthesis pathway (Figure 2A of the main text). The tubulin gene *pTUB22* encodes the β-tubulin protein (Figure 2B). The OAR, KCS and CYP86 represent the 3-oxoacyl-[acyl-carrier protein] reductase, 3-ketoacyl-CoA synthase and long-chain fatty acid omega-monooxygenase respectively, which all participate in the cutin, suberin and wax biosynthesis pathway (Figure 3A,C,D). SATR, Salt-Alkali Tolerant Rice.

Table S6. Primers and corresponding gene annotations for qRT-PCR analysis in this study

Gene ID	5'-primer	3'-primer	Gene symbol	Description
LOC_Os03g52630	ACCTGGGTGGTATTGTG-GACCT	TGATGCAC-CTCTGTGATGAGCG	*OsGH9A3*	Endoglucanase, putative, expressed
LOC_Os03g55070	CACGGTGTCGGGCAAGAA-GATC	CCTGCGGGTCG-TAGATGCTGAT		UDP-glucose 6-dehydrogenase, putative, expressed
LOC_Os03g55290	TCTCCTCCTGCTCCTGCTCTTG	GCACACGCCGCAG-TACTTGA	*OsGASR1*	GASR3-Gibberellin-regulated GASA/GAST/Snakin family protein precursor, expressed
LOC_Os07g41360	CGACGATATGTGGGCAG-GATGG	AGGGACGCAGACTG-GAAGAAGG	*OsUAM3*	Alpha-1,4-glucan-protein synthase, putative, expressed
LOC_Os07g43990	ATTGCGAC-CAATGAGCCGTTCT	CCTCCCACCAC-TTAGCGTTGTC		Expressed protein
LOC_Os07g44070	CGTGAAGTGCCTT-GCTGATGCT	TCCATGCGG-GAGGTACAAGACC		Pectinacetylesterase domain containing protein, expressed
LOC_Os07g47700	ACGGTGTACGGCGACGG-TAA	CCTTGGCAAGCTCCAG-CATTGT	*OsUXS6*	NAD dependent epimerase/dehydratase family domain containing protein, expressed
LOC_Os08g23810	GCCGAGCTGCCAAGGCAATT	GCCAGTGATAGCG-GAAGCCAAT		Enoyl-acyl-carrier-protein reductase NADH, chloroplast precursor, expressed

Table S7. Statistics analysis of the major agronomic traits of WT-C, *mq2*-C, WT-T and *mq2*-T plants

Major agronomic traits	Control		$NaHCO_3$ treatment		Comparison group 1	Comparison group 2
	WT-C	*mq2*-C	WT-T	*mq2*-T	WT-C / *mq2*-C	WT-T / *mq2*-T
Plant height (cm)	99.37 ± 4.40^{a}	65.72 ± 3.61^{c}	82.23 ± 2.85^{b}	50.00 ± 5.63^{d}	-33.86%	-39.20%
Total aboveground dry weight per plant (g)	29.39 ± 7.84^{a}	7.27 ± 1.99^{c}	16.23 ± 1.83^{b}	3.89 ± 1.15^{c}	-75.26%	-76.06%
Grain yield per plant (g)	13.75 ± 3.36^{a}	2.62 ± 0.68^{c}	6.23 ± 0.78^{b}	0.39 ± 0.14^{d}	-80.95%	-93.74%
Panicle number per plant	15.63 ± 5.45^{a}	8.88 ± 3.48^{b}	6.38 ± 1.41^{bc}	3.00 ± 1.6^{c}	-43.20%	-52.94%
Filled grain number per panicle	36.19 ± 3.49^{b}	15.12 ± 1.90^{c}	45.75 ± 8.21^{a}	9.91 ± 4.21^{c}	-58.22%	-78.34%
1000-grain weight (g)	23.78 ± 0.24^{a}	18.61 ± 0.26^{c}	22.76 ± 0.12^{b}	16.69 ± 0.19^{d}	-21.77%	-26.68%
Grain length (mm)	6.84 ± 0.03^{b}	7.04 ± 0.06^{a}	6.63 ± 0.04^{c}	6.58 ± 0.03^{c}	+3.02%	-0.86%
Grain width (mm)	3.38 ± 0.00^{a}	2.76 ± 0.00^{c}	3.27 ± 0.02^{b}	2.67 ± 0.02^{d}	-18.40%	-18.24%
Grain thickness (mm)	2.27 ± 0.18^{a}	2.05 ± 0.16^{b}	2.22 ± 0.07^{a}	2.00 ± 0.12^{b}	-9.71%	-9.52%
Panicle length (cm)	12.10 ± 0.35^{b}	13.41 ± 1.38^{a}	12.85 ± 0.65^{ab}	13.52 ± 1.16^{a}	+10.82%	+5.23%
Primary branch number per panicle	5.52 ± 0.33^{b}	3.73 ± 0.57^{c}	7.25 ± 0.72^{a}	4.11 ± 0.14^{c}	-32.40%	-43.27%
Second branch number per panicle	4.01 ± 0.75^{b}	0.98 ± 0.40^{c}	4.83 ± 0.74^{a}	0.30 ± 0.43^{d}	-75.64%	-93.70%
Total grain number per panicl	39.58 ± 3.76^{b}	20.89 ± 3.52^{c}	52.89 ± 6.48^{a}	23.44 ± 1.53^{c}	-47.21%	-55.67%
Seed setting rate (%)	91.50 ± 3.30^{a}	73.08 ± 7.22^{b}	86.10 ± 6.27^{a}	42.24 ± 17.15^{c}	-20.14%	-50.94%
Grain length/width ratio	2.03 ± 0.01^{c}	2.57 ± 0.03^{a}	2.04 ± 0.00^{c}	2.47 ± 0.01^{b}	+26.53%	+21.54%
Grain length/thickness ratio	3.03 ± 0.26^{c}	3.46 ± 0.31^{a}	3.00 ± 0.10^{c}	3.29 ± 0.19^{b}	+14.11%	+9.81%

Data of the major agronomic traits of WT-C, *mq2*-C, WT-T and *mq2*-T plants is given as mean ± SD. The different superscript letter in each row indicates the significant difference at $P < 0.05$ level obtained by one-way analysis of variance (ANOVA) with the Duncan's new multiple range test. The data in comparison group 1 indicates the percentage of decreasing or increasing percentage of the mean of each trait in *mq2*-C plants to WT-C plants {[(*mq2*-C-WT-C)/WT-C]×%} under controlled growth conditions without $NaHCO_3$ treatment. The data in comparison group 2 indicates the percentage of decreasing or increasing percentage of the mean of each trait in *mq2*-T plants to WT-T plants {[(*mq2*-T-WT-T)/WT-T]×%} under $NaHCO_3$ treatment (approximately 8‰ $NaHCO_3$ in the soil). WT, the wild-type plants (Kitaake) harboring the functional *OsCSLD4* gene; *mq2*, the *OsCSLD4* function-disrupted mutant of Kitaake.

Supplementary References

1. Xie, G.; Yang, B.; Xu, Z.; Li, F.; Guo, K.; Zhang, M.; Wang, L.; Zou, W.; Wang, Y.; Peng, L., Global identification of multiple OsGH9 family members and their involvement in cellulose crystallinity modification in rice. *PLoS One* **2013,** 8, (1), e50171.
2. Todaka, D.; Nakashima, K.; Maruyama, K.; Kidokoro, S.; Osakabe, Y.; Ito, Y.; Matsukura, S.; Fujita, Y.; Yoshiwara, K.; Ohme-Takagi, M.; Kojima, M.; Sakakibara, H.; Shinozaki, K.; Yamaguchi-Shinozaki, K., Rice phytochrome-interacting factor-like protein OsPIL1 functions as a key regulator of internode elongation and induces a morphological response to drought stress. *Proceedings of the National Academy of Sciences of the United States of America* **2012,** 109, (39), 15947-15952.
3. Wu, B.; Zhang, B.; Dai, Y.; Zhang, L.; Shang-Guan, K.; Peng, Y.; Zhou, Y.; Zhu, Z., *Brittle culm15* encodes a membrane-associated chitinase-like protein required for cellulose biosynthesis in rice. *Plant Physiology* **2012,** 159, (4), 1440-1452.
4. Leng, Y.; Yang, Y.; Ren, D.; Huang, L.; Dai, L.; Wang, Y.; Chen, L.; Tu, Z.; Gao, Y.; Li, X.; Zhu, L.; Hu, J.; Zhang, G.; Gao, Z.; Guo, L.; Kong, Z.; Lin, Y.; Qian, Q.; Zeng, D., A rice *PECTATE LYASE-LIKE* gene is required for plant growth and leaf senescence. *Plant Physiology* **2017,** 174, (2), 1151-1166.
5. Wang, D.; Qin, Y.; Fang, J.; Yuan, S.; Peng, L.; Zhao, J.; Li, X., A missense mutation in the zinc finger domain of OsCESA7 deleteriously affects cellulose biosynthesis and plant growth in rice. *PLoS One* **2016,** 11, (4), e0153993.
6. Takeda, Y.; Koshiba, T.; Tobimatsu, Y.; Suzuki, S.; Murakami, S.; Yamamura, M.; Rahman, M. M.; Takano, T.; Hattori, T.; Sakamoto, M.; Umezawa, T., Regulation of *CONIFERALDEHYDE 5-HYDROXYLASE* expression to modulate cell wall lignin structure in rice. *Planta* **2017,** 246, (2), 337-349.

7. Zou, H.; Wenwen, Y.; Zang, G.; Kang, Z.; Zhang, Z.; Huang, J.; Wang, G., *OsEXPB2*, a β-expansin gene, is involved in rice root system architecture. *Molecular Breeding* **2015,** 35, (1), 41.
8. Lee, S.-C.; Han, S.-K.; Kim, S.-R., Salt- and ABA-inducible *OsGASR1* is involved in salt tolerance. *Journal of Plant Biology* **2015,** 58, (2), 96-101.
9. Fang, L.; Zhao, F.; Cong, Y.; Sang, X.; Du, Q.; Wang, D.; Li, Y.; Ling, Y.; Yang, Z.; He, G., Rolling-leaf14 is a 2OG-Fe (II) oxygenase family protein that modulates rice leaf rolling by affecting secondary cell wall formation in leaves. *Plant Biotechnology Journal* **2012,** 10, (5), 524-532.
10. Kim, C. M.; Park, S. H.; Je, B. I.; Park, S. H.; Park, S. J.; Piao, H. L.; Eun, M. Y.; Dolan, L.; Han, C. D., *OsCSLD1*, a cellulose synthase-like D1 gene, is required for root hair morphogenesis in rice. *Plant Physiology* **2007,** 143, (3), 1220-1230.
11. Wang, F. J.; Zhu, C., Heterologous expression of a rice syntaxin-related protein KNOLLE gene (*OsKNOLLE*) in yeast and its functional analysis in the role of abiotic stress. *HEREDITAS (Beijing)* **2011,** 33, (11), 1251-1257.
12. Singh.; Kumar.; Pareek.; Sopory.; Singla-Pareek., Overexpression of rice CBS domain containing protein improves salinity, oxidative, and heavy metal tolerance in transgenic tobacco. *Molecular Biotechnology* **2012,** 52, (3), 205-216.
13. Seo, J. S.; Joo, J.; Kim, M. J.; Kim, Y. K.; Nahm, B. H.; Song, S. I.; Cheong, J. J.; Lee, J. S.; Kim, J. K.; Choi, Y. D., OsbHLH148, a basic helix-loop-helix protein, interacts with OsJAZ proteins in a jasmonate signaling pathway leading to drought tolerance in rice. *The Plant Journal* **2011,** 65, (6), 907-921.
14. Tuteja, N.; Sahoo, R. K.; Garg, B.; Tuteja, R., OsSUV3 dual helicase functions in salinity stress tolerance by maintaining photosynthesis and antioxidant machinery in rice (*Oryza sativa* L. cv. IR64). *The Plant Journal* **2013,** 76, (1), 115-127.
15. Zhong, R.; Wang, Y.; Gai, R.; Xi, D.; Mao, C.; Ming, F., Rice SnRK protein kinase *OsSAPK8* acts as a positive regulator in abiotic stress responses. *Plant Science* **2020,** 292, 110373.
16. Chapagain, S.; Park, Y. C.; Kim, J. H.; Jang, C. S., Oryza sativa salt-induced RING E3 ligase 2 (*OsSIRP2*) acts as a positive regulator of transketolase in plant response to salinity and osmotic stress. *Planta* **2018,** 247, (4), 925-939.
17. Zhao, J.; Li, M.; Gu, D.; Liu, X.; Zhang, J.; Wu, K.; Zhang, X.; Teixeira da Silva, J. A.; Duan, J., Involvement of rice histone deacetylase HDA705 in seed germination and in response to ABA and abiotic stresses. *Biochemical and Biophysical Research Communications* **2016,** 470, (2), 439-444.
18. Giri, J.; Vij, S.; Dansana, P. K.; Tyagi, A. K., Rice A20/AN1 zinc-finger containing stress-associated proteins (SAP1/11) and a receptor-like cytoplasmic kinase (OsRLCK253) interact via A20 zinc-finger and confer abiotic stress tolerance in transgenic *Arabidopsis* plants. *New Phytologist* **2011,** 191, (3), 721-732.
19. Wu, T. M.; Lin, W. R.; Kao, C. H.; Hong, C. Y., Gene knockout of *glutathione reductase 3* results in increased sensitivity to salt stress in rice. *Plant Molecular Biology* **2015,** 87, (6), 555-564.
20. Alfatih, A.; Wu, J.; Jan, S. U.; Zhang, Z. S.; Xia, J. Q.; Xiang, C. B., Loss of rice *PARAQUAT TOLERANCE 3* confers enhanced resistance to abiotic stresses and increases grain yield in field. *Plant, Cell and Environment* **2020,** 43, (11), 2743-2754.
21. Li, L. H.; Lv, M. M.; Li, X.; Ye, T. Z.; He, X.; Rong, S. H.; Dong, Y. L.; Guan, Y.; Gao, X. L.; Zhu, J. Q.; Xu, Z. J., The rice *OsDUF810* family: OsDUF810.7 may be Involved in the tolerance to salt and drought. *Molecular Biology* **2018,** 52, (4), 489-496.
22. Islam, M. O.; Kato, H.; Shima, S.; Tezuka, D.; Matsui, H.; Imai, R., Functional identification of a rice trehalase gene involved in salt stress tolerance. *Gene* **2019,** 685, 42-49.
23. Sharma, R.; Sahoo, A.; Devendran, R.; Jain, M., Over-expression of a rice tau class glutathione s-transferase gene improves tolerance to salinity and oxidative stresses in Arabidopsis. *PLoS One* **2014,** 9, (3), e92900.
